# Stereoselective Synthesis of *meso*- and l,l-Diaminopimelic Acids from Enone-Derived α-Amino Acids

**DOI:** 10.1021/acs.joc.4c00916

**Published:** 2024-07-02

**Authors:** Sineenard Songsri, Holly McErlain, Andrew Sutherland

**Affiliations:** School of Chemistry, The Joseph Black Building, University of Glasgow, Glasgow G12 8QQ, United Kingdom

## Abstract

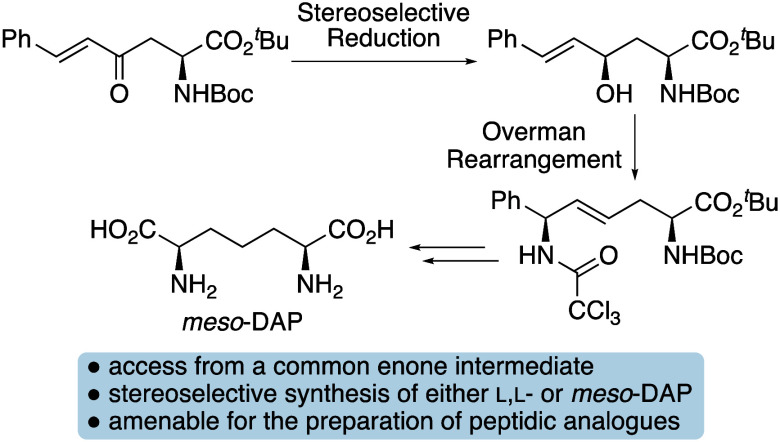

The stereoselective synthesis of *meso*-diaminopimelic acid (*meso*-DAP), the key cross-linking amino acid of the peptidoglycan cell wall layer in Gram-negative bacteria, and its biological precursor, l,l-DAP, is described. The key step involved stereoselective reduction of a common enone-derived amino acid by substrate- or reagent-based control. Overman rearrangement of the resulting allylic alcohols, concurrent alkene hydrogenation and trichloroacetamide reduction, and subsequent ruthenium-catalyzed arene oxidation completed the synthesis of each stereoisomer. The synthetic utility of this approach was demonstrated with the efficient preparation of an l,l-DAP-derived dipeptide.

Bacterial cell wall biosynthesis has long been a target for the development of antibiotics; however, the emergence of bacterial resistance to traditional antibiotics such as β-lactams and glycopeptides has led to the investigation of other processes that construct the bacterial cell wall.^1^ The peptidoglycan layer (**1**) is an important component of cell walls in most bacteria and provides cell integrity by withstanding internal osmotic pressure.^1^ In Gram-negative bacteria, the key cross-linking amino acid is *meso*-diaminopimelic acid (DAP), which connects the alternating chains of *N*-acetyl glucosamine and *N*-acetyl muramic acid through a short peptide (Figure 1a).^2,3^ In Gram-positive bacteria, the amino acid that cross-links the peptidoglycan layer is l-lysine, which is a product of *meso*-DAP biosynthesis (Figure 1b).^4^ This pathway utilizes l-aspartate semialdehyde (**2**) and pyruvate, which are converted to l,l-DAP (**3**) via picolinate intermediates. The cofactor free enzyme, DAP epimerase converts l,l-DAP (**3**) to *meso*-DAP (**4**), which is then used in cell wall biosynthesis. In Gram-positive bacteria, decarboxylation of *meso*-DAP (**4**) generates l-lysine (**5**). As *meso*-DAP (**4**) and l-lysine (**5**) are crucial to maintain bacterial cell wall integrity and their biosynthesis is not present in mammals, the inhibition of the DAP enzymes has been widely studied as an approach to develop novel antibacterials.^5^

**Figure 1:**
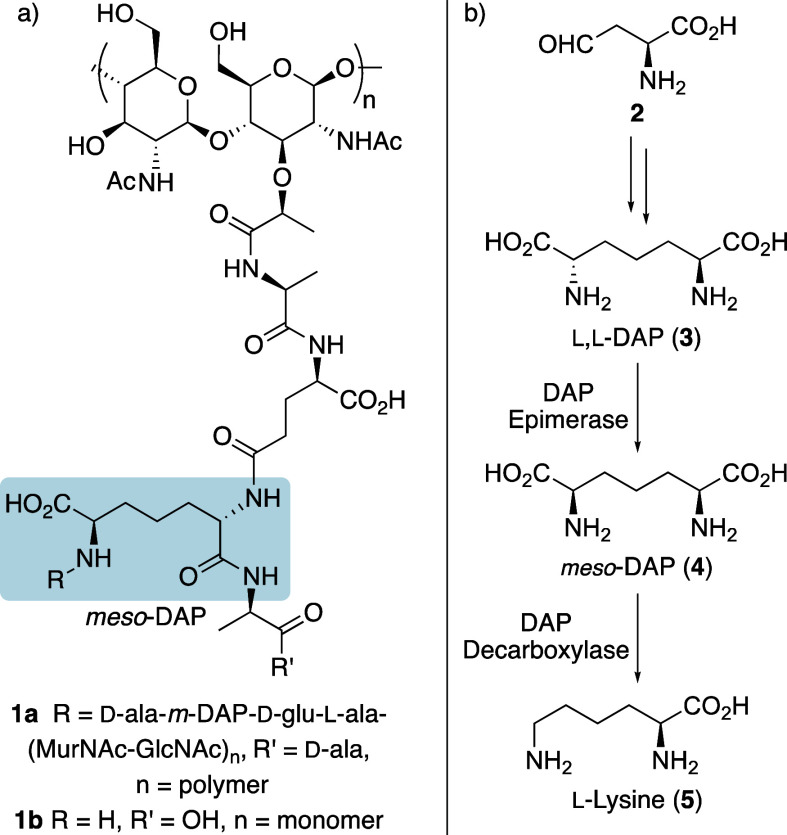
(a) Structure of peptidoglycan in Gram-negative bacteria. (b) Biosynthesis of l-lysine via *meso*-DAP.

To investigate and then inhibit the DAP enzymes has required significant synthetic efforts and in particular, the stereoselective synthesis of *meso*-DAP and l,l-DAP analogues.^5,6^ A variety of strategies have been used including chiral pool and chiral auxiliary based approaches.^7^ Methods that generate these compounds with orthogonal amine and carboxylic acid protecting groups for selective peptide synthesis have also been developed.^8^ For example, Vederas and co-workers reported the synthesis of DAP compounds by radical-based decarboxylation and addition reactions of functionalized aspartate and glutamate derivatives,^9^ and then used the products to study DAP analogues of the lantibiotic peptide lactocin S.^9b^ Another general approach has been the development of asymmetric methods that produce optically active secondary alcohols as key synthetic intermediates. For example, Roberts and Chan described the synthesis of *meso*-DAP (**4**) by stereoselective reduction of an α-keto ester using (*R*)-(+)-alpine-borane (Scheme 1a),^10^ while the Walsh group reported the preparation of an l,l-DAP analogue by asymmetric double addition of vinylzinc reagents with benzaldehyde in the presence of an isoborneol-based amino alcohol ligand (Scheme 1b).^11^

**Scheme 1:**
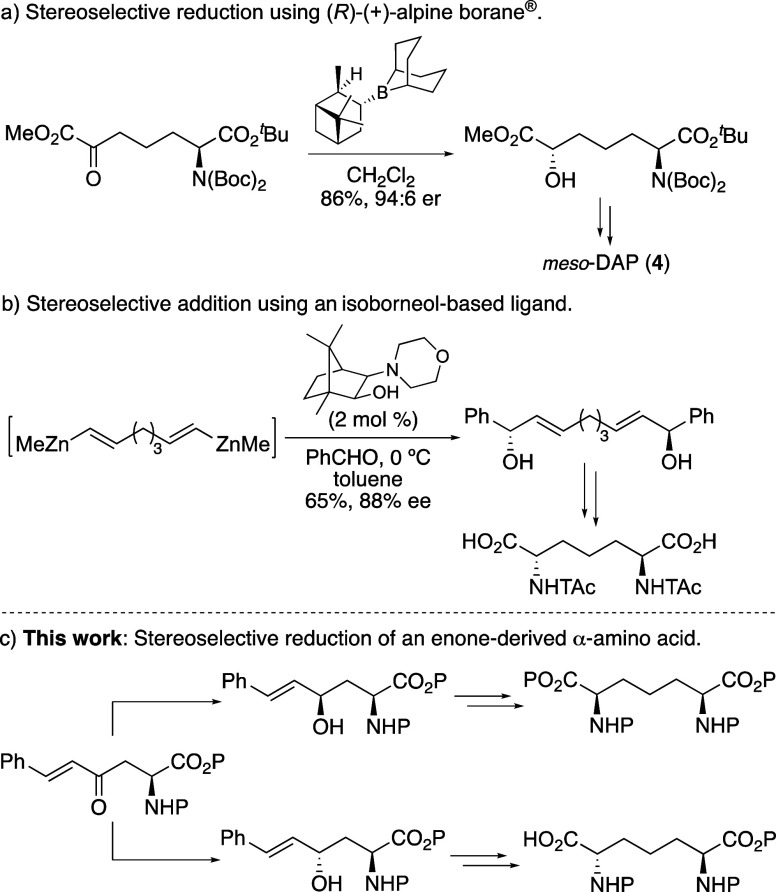
Various Approaches for the Stereoselective Synthesis of Diaminopimelic Acid Compounds

In 2009, we reported the general synthesis of enone-derived α-amino acids via the Horner-Wadsworth-Emmons (HWE) reaction of an l-aspartic acid-derived phosphonate ester with various aldehydes.^12^ Subsequently, these compounds were found to be excellent synthetic intermediates for a range of applications. Intramolecular conjugate addition reactions allowed the stereoselective synthesis of pipecolic acids,^13,14^ while cycloaddition processes led to the preparation of fluorescent unnatural α-amino acids.^15^ To further explore the synthetic utility of enone-derived α-amino acids and inspired by the work of the Roberts and Walsh groups,^10,11^ we proposed that these could undergo stereoselective reduction to give the corresponding allylic alcohol (Scheme 1c). It was believed that either stereoisomer could be accessed via a single enone intermediate and following stereoselective reduction, used to prepare suitably protected *meso*-DAP or l,l-DAP derivatives, as well as peptide analogues. Here, we report a new approach for the efficient synthesis of enone-derived α-amino acids, followed by stereoselective reduction using either substrate- or reagent-based control. We also describe the application of the resulting allylic alcohols for the synthesis of *meso*-DAP and l,l-DAP derivatives using an Overman rearrangement and a ruthenium-catalyzed arene oxidation as the key steps.

The initial aim of this project was the efficient synthesis of an enone-derived α-amino acid, followed by stereoselective reduction of the ketone to access the corresponding allylic alcohol. Jackson and co-workers previously reported that 4-oxo α-amino acid derivatives can undergo stereoselective substrate-controlled reduction using bulky reagents such as L-selectride.^16^ Although these reactions resulted in selective *syn*-reduction, *in situ* intramolecular cyclization of the resulting alcohol with the α-ester gave the corresponding lactone as the final product. To prevent lactonization, it was proposed that this could be achieved using a bulky α-*tert*-butyl ester. Precedent for this was provided by Rudolph and co-workers who showed that during their stereoselective synthesis of (2*S*,4*R*)-4-hydroxyornithine, L-selectride reduction of a 4-oxo α-amino acid derivative with a *tert*-butyl ester generated the *erythro*-4-hydroxy product in high diastereoselectivity (85:15) with no lactonization.^17^ Thus, for this current work, commercially available Boc-l-aspartic acid α-*t*-butyl ester (**6**) was proposed as the starting material and used for a new three-step synthesis of enone-derived α-amino acids (Scheme 2). Aspartic acid derivative **6** was converted to Weinreb amide **7** in quantitative yield using *N*,*O*-dimethyl hydroxylamine hydrochloride in the presence of 2-(1*H*-benzotriazole-1-yl)-1,1,3,3-trimethylaminium tetrafluoroborate (TBTU) and HOBt (Scheme 2).^18^ Reaction of Weinreb amide **7** with the anion of dimethyl methylphosphonate resulted in a regioselective reaction and gave β-ketophosphonate ester **8** in 72% yield. Horner-Wadsworth-Emmons reaction of **8** with benzaldehyde under basic conditions gave *E*-enone **9** as the sole product in 91% yield. This novel approach for the preparation of an enone-derived α-amino acid using a Weinreb amide intermediate allowed a scalable, regioselective and efficient synthesis in only three steps. Substrate-controlled stereoselective reduction using L-selectride was then investigated. Reaction of L-selectride with enone **9** at −78 °C proceeded cleanly and from the ^1^H NMR spectrum of the crude reaction mixture showed an 8:1 ratio of two diastereomeric products. Separation by column chromatography allowed isolation of the major product, (2*S*,4*R*)-allylic alcohol **10** in 75% yield. Allylic alcohol **10** was then used to complete the synthesis of fully protected *meso*-DAP analogue **15** and *meso*-DAP (**4**) (Scheme 2). Reaction of **10** with trichloroacetonitrile and catalytic DBU, followed by Overman rearrangement of resulting allylic trichloroacetimidate **11** under thermal conditions gave allylic trichloroacetamide **12** in 77% yield over the two steps.^19^ It was assumed that rearrangement proceeded with retention of configuration as only one diastereomer was observed by NMR spectroscopy. Hydrogenation using palladium on carbon under basic conditions led to reduction of the alkene and trichloroacetamide to give saturated *N*-acetyl derivative **13** in 95% yield. For the final stage of the synthesis, which required arene oxidation, methods reported by the Sharpless and Martín groups that use ruthenium catalysis and sodium periodate as an *in situ* oxidant were considered.^20^ Initial attempts at oxidation of **13** using RuCl_3_.3H_2_O (20 mol %) under standard conditions led to moderate conversions (50–60%). For this reason and the increasing inaccessibility of carbon tetrachloride, it was decided to optimize the oxidation of **13**. Initially, a range of alternative solvents for CCl_4_ were screened and the highest conversions (>60%) were observed using dichloroethane (DCE). The other issue found with this transformation was catalyst inactivation after 24 h. Therefore, a second batch of RuCl_3_.3H_2_O (20 mol %) was added leading to the highest conversion of 79%. The resulting carboxylic acid **14** was then isolated as the methyl ester by reaction with trimethylsilyldiazomethane and methanol. This gave fully protected *meso*-DAP analogue **15** in 65% yield over the two steps. This completed the nine-step synthesis of *meso*-DAP analogue **15** in 22% overall yield using a substrate-controlled stereoselective reduction as the key step. To confirm the stereoselective outcome of the L-selectride reduction of enone **9**, carboxylic acid **14** was also deprotected to give *meso*-DAP (**4**). Acid-mediated removal of the protecting groups and recrystallization from ethanol gave the dihydrochloride salt of *meso*-DAP (**4**) in 79% yield (51% from **13**). The physical and spectroscopic data of our material was in agreement with literature values,^21^ thereby confirming *erthyro*-diastereomer **10** as the product from L-selectride reduction of enone **9**.

**Scheme 2:**
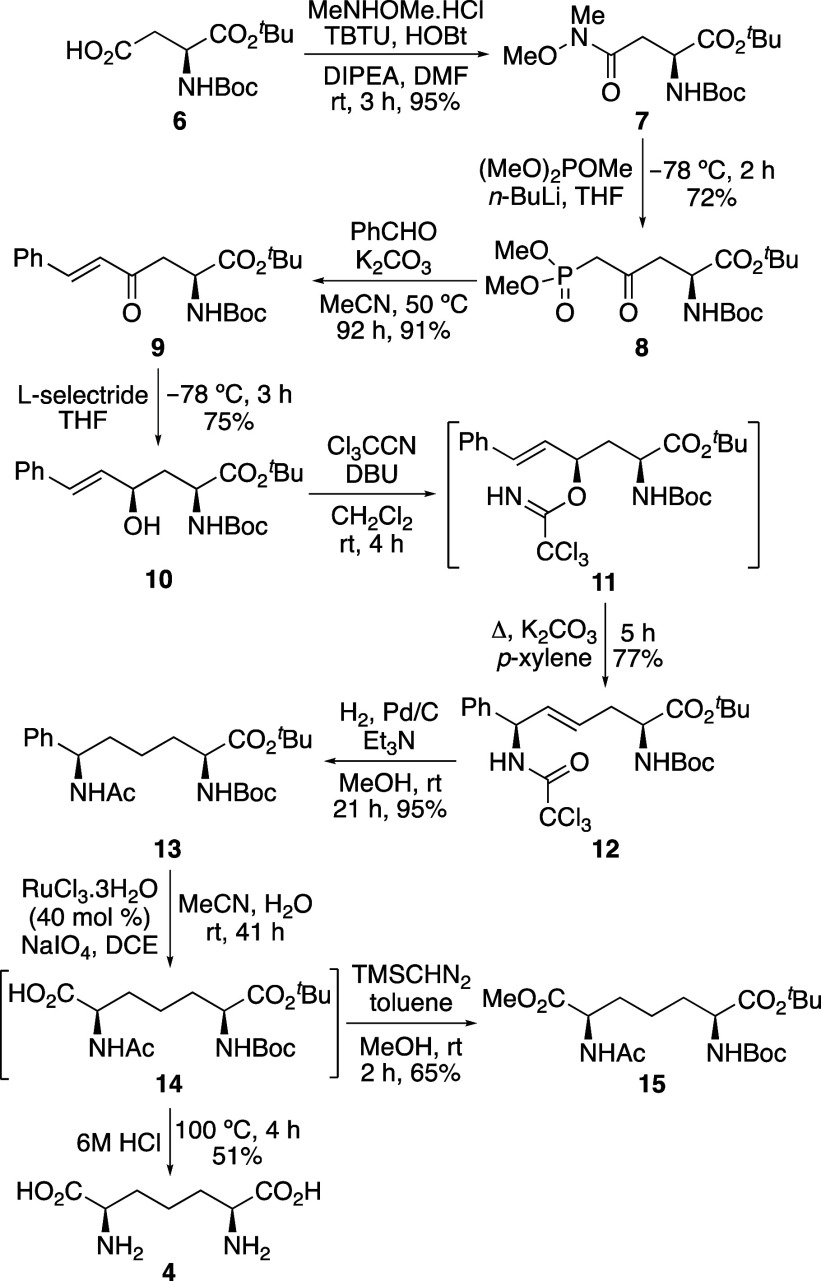
Stereoselective Synthesis of *meso*-DAP **4** Isolated yields.

To overcome the chiral bias of enone-derived α-amino acid **9** and access the l,l-DAP series of compounds, a reagent-controlled reduction was explored. The commercially available CBS-oxazaborolidine catalysts have been shown to perform clean 1,2-reduction of enones to give the corresponding allylic alcohols with high stereoselectivity and thus, (*R*)-CBS-Me reduction of enone **9** was investigated (Scheme 3).^22^ Initially, the use of stoichiometric amounts of (*R*)-CBS-Me in the presence of borane-THF gave *threo*-(2*S*,4*S*)-diastereomer **16** as the sole product in 87% yield. The stereochemical outcome was confirmed by comparison with the two diastereomeric products from L-selectride reduction of enone **9**, in which **16** was found to be the minor diastereomer. The use of catalytic (*R*)-CBS-Me (10 mol %) was also investigated and while this gave a lower yield (70%), allylic alcohol **16** was the only diastereomer generated. Allylic alcohol **16** was then converted to fully protected l,l-DAP analogue **20** using the same approach as that described for *meso*-DAP compound **15**. Allylic imidate formation and Overman rearrangement gave allylic trichloroacetamide **18** in 66% yield. Reduction of the alkene and trichloroacetyl group followed by ruthenium(III)-mediated arene oxidation and esterification gave l,l-DAP compound **20** in similar yields as described for **15**.

**Scheme 3:**
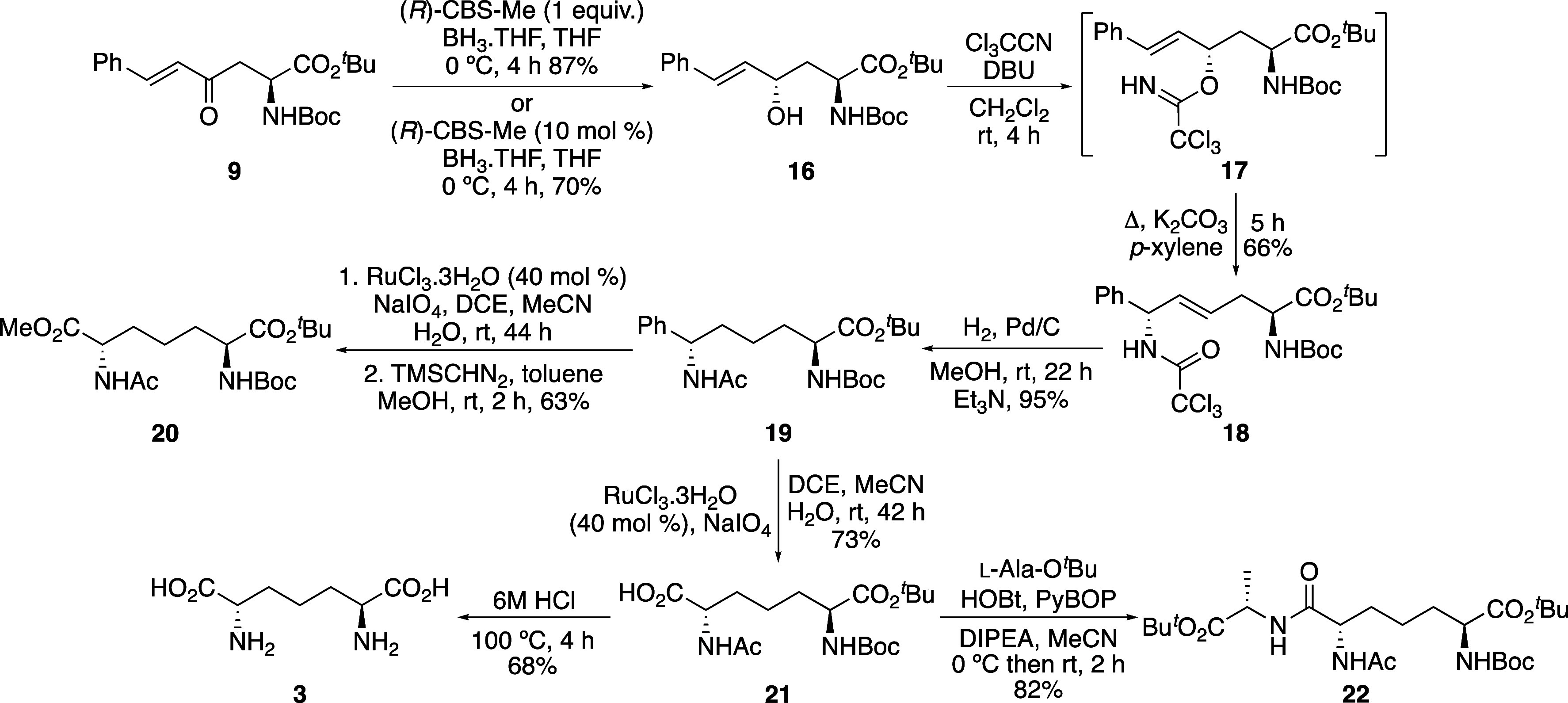
Stereoselective Synthesis of l,l-DAP (**3**) and Dipeptide **22** Isolated yields.

During arene oxidation of **19**, it was found that carboxylic acid **21** was formed cleanly and could be isolated in 73% yield (Scheme 3). In a similar manner to the *meso*-DAP route, this material was used to further confirm the stereochemical outcome of (*R*)-CBS-Me reduction of enone **9** by conversion to l,l-DAP (**3**). Acid-mediated removal of the protecting groups of **21** gave l,l-DAP (**3**) as the dihydrochloride salt in 68% yield. The optical rotation and spectroscopic data of the material matched literature values,^23^ thereby confirming *threo*-diastereomer **16** as the product from (*R*)-CBS-Me reduction of enone **9**. Carboxylic acid **21** was also used as an intermediate for the synthesis of an l,l-DAP-containing dipeptide. Antibacterial dipeptides containing the DAP skeleton and l-alanine have been isolated from *Micromonospora chalcea*.^24^ Thus, it was decided to couple carboxylic acid **21** with an alanine derivative. Coupling of **21** with l-alanine *tert*-butyl ester in the presence of HOBt and PyBOP gave l,l-DAP-l-alanine dipeptide **22** in 82% yield. The straightforward nature and efficiency of this process demonstrates the compatibility of this synthetic route for the preparation of DAP-containing dipeptides.

In summary, a stereoselective approach for the preparation of *meso*-DAP and l,l-DAP compounds has been developed from enone-derived α-amino acids. Substrate-controlled reduction using L-selectride gave an *erythro*-(2*S*,4*R*)-allylic alcohol in high diastereoselectivity, while the use of a reagent-controlled reduction using (*R*)-CBS-Me gave the *threo*-(2*S*,4*S*)-diastereomer as the sole product. Both allylic alcohols were excellent substrates for Overman rearrangement, allowing the installation of the second amino group in a stereocontrolled manner. Ruthenium-mediated arene oxidation and esterification completed the nine-step synthesis of fully protected *meso*-DAP and l,l-DAP compounds. Alternatively, deprotection permitted efficient access to both *meso*-DAP (**4**) and l,l-DAP (**3**). The synthetic utility of this approach was demonstrated with the synthesis of a DAP-containing dipeptide. This aspect of the work is currently being explored for the preparation of novel DAP-derived peptides.

## Experimental Section

All reagents and starting materials were obtained from commercial sources and used as received. Compound **7** were prepared as previously reported.^18b^ Reactions were performed open to air unless otherwise mentioned. All reactions performed at elevated temperatures were heated using an oil bath. Brine refers to a saturated aqueous solution of sodium chloride. Flash column chromatography was performed using silica gel 60 (40–63 μm). Aluminum-backed plates precoated with silica gel 60F_254_ were used for thin layer chromatography and were visualized with a UV lamp or by staining with potassium permanganate or ninhydrin. ^1^H NMR spectra were recorded on a NMR spectrometer at 400 MHz and data are reported as follows: chemical shift in ppm relative to tetramethylsilane or the to the solvent as internal standard (CHCl_3_, δ 7.26 ppm; H_2_O, δ 4.79 ppm; CH_3_OH, δ 3.31 ppm), multiplicity (s = singlet, d = doublet, t = triplet, q = quartet, m = multiplet or overlap of nonequivalent resonances, integration). The abbreviation br s refers to broad singlet. ^13^C{^1^H} NMR spectra were recorded on a NMR spectrometer at 101 MHz and data are reported as follows: chemical shift in ppm relative to tetramethylsilane or the solvent as internal standard (CDCl_3_, δ 77.0 ppm; CD_3_OD, δ 49.0 ppm). Infrared spectra were recorded on a FTIR spectrometer; wavenumbers are indicated in cm^–1^. Mass spectra were recorded using electrospray (ESI) or atmospheric pressure chemical ionization (APCI) techniques on a quadrupole time-of-flight (Q-TOF) mass spectrometer. Melting points are uncorrected. Optical rotations were determined as solutions irradiating with the sodium D line (λ = 589 nm) using a polarimeter. [α]_D_ values are given in units 10^–1^ deg cm^–1^ g^–1^.

### *tert*-Butyl (2*S*)-2-(*tert*-Butoxycarbonylamino)-5-(dimethoxyphosphoryl)-4-oxopentanoate (**8**)

In an oven-dried flask, a solution of dimethyl methylphosphonate (1.22 mL, 11.3 mmol) in anhydrous THF (20 mL) was cooled to −78 °C under argon. *n*-Butyl lithium (2.5 M in hexane, 4.50 mL, 11.3 mmol) was added dropwise and the mixture was stirred for 1 h. In a separate oven-dried flask, a solution of *tert*-butyl (2*S*)-2-(*tert*-butoxycarbonylamino)-4-[methoxy(methyl)amino]-4-oxobutanoate (**7**) (1.09 g, 3.23 mmol) in anhydrous THF (40 mL) was cooled to −78 °C under argon. The dimethyl methylphosphonate/*n*-butyl lithium solution was then cannulated into the flask containing the Weinreb amide/THF solution and stirred for 2 h to give a yellow solution. The reaction was quenched with saturated aqueous ammonium chloride solution (2 mL), allowed to warm to room temperature and concentrated *in vacuo*. The resultant residue was diluted with ethyl acetate (50 mL), washed with water (2 × 50 mL) and then brine (50 mL), dried (MgSO_4_), filtered and concentrated *in vacuo*. Purification by flash column chromatography eluting with 75% ethyl acetate in dichloromethane gave *tert*-butyl (2*S*)-5-(dimethoxyphosphoryl)-4-oxo-2-(tritylamino)pentanoate (**8**) as a white solid (0.940 g, 72%). Mp 72–75 °C; IR (neat) 3290, 2976, 1713, 1612, 1491, 1362, 1246, 1153, 851, 751 cm^–1^; [α]_D_^22^ +23.7 (*c* 1.0, CHCl_3_); ^1^H NMR (400 MHz, CDCl_3_): δ 5.45 (d, *J* = 8.4 Hz, 1H), 4.37 (dt, *J* = 8.4, 4.5 Hz, 1H), 3.81 (s, 3H), 3.78 (s, 3H), 3.27 (dd, *J* = 18.3, 4.5 Hz, 1H), 3.17–3.04 (m, 3H), 1.45 (s, 9H), 1.44 (s, 9H); ^13^C{^1^H} NMR (101 MHz, CDCl_3_): δ 199.8 (d, ^2^*J*_C–P_ = 6.3 Hz), 170.0, 155.6, 82.3, 79.8, 53.2 (d, ^2^*J*_C–O–P_ = 3.2 Hz), 53.1 (d, ^2^*J*_C–O–P_ = 3.2 Hz), 50.1, 46.1 (d, ^3^*J*_C–O–P_ = 1.2 Hz), 41.4 (d, ^1^*J*_C–P_ = 128.4 Hz), 28.3, 27.8; MS (ESI) *m*/*z* 418 (M + Na^+^, 100); HRMS (ESI) *m*/*z*: [M + Na]^+^ Calcd for C_16_H_30_NO_8_PNa 418.1601; Found 418.1603.

### *tert*-Butyl (2*S*,5*E*)-2-(*tert*-Butoxycarbonylamino)-4-oxo-6-phenylhex-5-enoate (**9**)

In an oven-dried flask, *tert*-butyl (2*S*)-2-(*tert*-butoxycarbonylamino)-5-(dimethoxyphosphoryl)-4-oxopentanoate (**8**) (0.743 g, 1.88 mmol) was dissolved in anhydrous acetonitrile (19 mL) under argon. Anhydrous potassium carbonate (0.286 g, 2.07 mmol) was added and the mixture was stirred for 0.5 h. Benzaldehyde (0.390 mL, 3.84 mmol) was added to the suspension which was then heated to 50 °C and stirred for 92 h. The reaction mixture was cooled to room temperature and concentrated *in vacuo*. The resultant residue was dissolved in ethyl acetate (20 mL) and washed with water (2 × 15 mL) and then brine (15 mL), dried (MgSO_4_), filtered and concentrated *in vacuo*. Purification by flash column chromatography eluting with 15% ethyl acetate in hexane gave *tert*-butyl (2*S*,5*E*)-2-(*tert*-butoxycarbonylamino)-4-oxo-6-phenylhex-5-enoate (**9**) as an off-white solid (0.645 g, 91%). Mp 73–75 °C; IR (neat) 3338, 2979, 1711, 1666, 1611, 1496, 1367, 1154, 759 cm^–1^; [α]_D_^22^ +19.0 (*c* 0.1, CHCl_3_); ^1^H NMR (400 MHz, CDCl_3_): δ 7.60–7.53 (m, 3H), 7.43–7.38 (m, 3H), 6.72 (d, *J* = 16.3 Hz, 1H), 5.54 (d, *J* = 8.6 Hz, 1H), 4.49 (dt, *J* = 8.6, 4.3 Hz, 1H), 3.38 (dd, *J* = 17.6, 4.3 Hz, 1H), 3.18 (dd, *J* = 17.6, 4.3 Hz, 1H), 1.45 (s, 9H), 1.44 (s, 9H); ^13^C{^1^H} NMR (101 MHz, CDCl_3_): δ 197.7, 170.4, 155.6, 143.6, 134.2, 130.8, 129.0, 128.4, 125.8, 82.0, 79.7, 50.3, 42.5, 28.3, 27.9; MS (ESI) *m*/*z* 398 (M + Na^+^, 100); HRMS (ESI) *m*/*z*: [M + Na]^+^ Calcd for C_21_H_29_NO_5_Na 398.1938; Found 398.1935.

### *tert*-Butyl (2*S*,4*R*,5*E*)-2-(*tert*-Butoxycarbonylamino)-4-hydroxy-6-phenylhex-5-enoate (**10**)

In an oven-dried flask, *tert*-butyl (2*S*,5*E*)-2-(*tert*-butoxycarbonylamino)-4-oxo-6-phenylhex-5-enoate (**9**) (0.12 g, 0.31 mmol) was dissolved in anhydrous THF (15 mL) under argon and cooled to at −78 °C. To this was added L-selectride (0.49 mL, 0.49 mmol, 1 M in THF) dropwise. The mixture was allowed to stir for 3 h before being quenched with an aqueous solution of ammonium chloride (5 mL). The reaction mixture was extracted with ethyl acetate (3 × 20 mL). The combined organic layers were washed with water (20 mL), brine (20 mL), dried (MgSO_4_), filtered and concentrated *in vacuo*. Purification by flash column chromatography on silica eluting with 50% diethyl ether in hexane gave *tert*-butyl (2*S*,4*R*,5*E*)-2-(*tert*-butoxycarbonylamino)-4-hydroxy-6-phenylhex-5-enoate (**10**) as a white solid (0.087 g, 75%). Mp 103–106 °C; IR (neat) 3361, 2978, 1698, 1496, 1367, 1250, 1153, 748 cm^–1^; [α]_D_^19^ +30.6 (*c* 0.1, CHCl_3_); ^1^H NMR (400 MHz, CDCl_3_): 7.40–7.20 (m, 5H), 6.61 (d, *J* = 15.9 Hz, 1H), 6.24 (dd, *J* = 15.9, 6.0 Hz, 1H), 5.35 (d, *J* = 6.8 Hz, 1H), 4.53–4.48 (m, 1H), 4.34–4.30 (m, 1H), 2.65 (br s, 1H), 2.19–2.10 (m, 1H), 1.95 (dt, *J* = 12.0, 8.0 Hz, 1H), 1.48 (s, 9H), 1.42 (s, 9H); ^13^C{^1^H} NMR (101 MHz, CDCl_3_): δ 171.8, 155.6, 136.7, 131.6, 130.1, 128.5, 127.7, 126.5, 82.2, 80.1, 70.0, 51.9, 40.3, 28.3, 28.0; MS (ESI) *m*/*z* 400 (M + Na^+^, 100); HRMS (ESI) *m*/*z*: [M + Na]^+^ Calcd for C_21_H_31_NO_5_Na 400.2094; Found 400.2085.

### *tert*-Butyl (2*S*,4*E*,6*R*)-2-(*tert*-Butoxycarbonylamino)-6-phenyl-6-(trichloromethylcarbonylamino)hex-4-enoate (**12**)

In an oven-dried flask, *tert*-butyl (2*S*,4*R*,5*E*)-2-(*tert*-butoxycarbonylamino)-4-hydroxy-6-phenylhex-5-enoate (**10**) (0.0921 g, 0.244 mmol) was dissolved in anhydrous dichloromethane (3.5 mL) under argon and cooled to 0 °C. To this solution was added trichloroacetonitrile (0.0370 mL, 0.369 mmol), followed by 1,8-diazabicyclo[5.4.0]undec-7-ene (0.0190 mL, 0.127 mmol). The reaction mixture was stirred at 0 °C for 1 h and then allowed to reach room temperature over 3 h. The mixture was filtered through a short pad of neutral alumina with dichloromethane (30 mL) and concentrated *in vacuo* to afford crude allylic trichloroacetimidate **11** as a yellow oil, which was used without further purification. Allylic trichloroacetimidate **11** was dissolved in anhydrous *p*-xylene (1.1 mL) under argon and transferred to an oven-dried microwave vial containing anhydrous potassium carbonate (0.00300 g, 0.0217 mmol). The tube was purged with argon, sealed and heated to 140 °C for 5 h. The reaction mixture was cooled to room temperature and concentrated *in vacuo*. Purification by flash column chromatography eluting with 10% ethyl acetate in hexane gave *tert*-butyl (2*S*,4*E*,6*R*)-2-(*tert*-butoxycarbonylamino)-6-phenyl-6-(trichloromethylcarbonylamino)hex-4-enoate (**12**) as a colorless oil (0.0978 g, 77%). IR (neat) 3323, 2926, 1698, 1504, 1367, 1153, 759 cm^–1^; [α]_D_^20^ +17.2 (*c* 0.5, CHCl_3_); ^1^H NMR (400 MHz, CDCl_3_): δ 7.40–7.28 (m, 5H), 6.96 (d, *J* = 7.7 Hz, 1H), 5.78 (dd, *J* = 15.4, 5.7 Hz, 1H), 5.64 (dt, *J* = 15.4, 8.0 Hz, 1H), 5.56–5.51 (m, 1H), 5.10 (d, *J* = 7.7 Hz, 1H), 4.30–4.23 (m, 1H), 2.63 (dt, *J* = 16.0, 8.0 Hz, 1H), 2.46 (dt, *J* = 16.0, 8.0 Hz, 1H), 1.44 (s, 9H), 1.43 (s, 9H); ^13^C{^1^H} NMR (101 MHz, CDCl_3_): δ 171.0, 161.0, 155.3, 139.2, 131.6, 129.1, 128.3, 127.0, 92.7, 82.4, 79.9, 56.5, 53.6, 35.8, 28.5, 28.2; MS (ESI) *m*/*z* 543 (M + Na^+^, 100); HRMS (ESI) *m*/*z*: [M + Na]^+^ Calcd for C_23_H_31_^35^Cl_3_N_2_O_5_Na 543.1191; Found 543.1173.

### *tert*-Butyl (2*S*,6*R*)-6-Acetamido-2-(*tert*-butoxycarbonylamino)-6-phenylhexanoate (**13**)

A stirred solution of *tert*-butyl (2*S*,4*E*,6*R*)-2-(*tert*-butoxycarbonylamino)-6-phenyl-6-(trichloromethylcarbonylamino)hex-4-enoate (**12**) (1.28 g, 2.45 mmol) in methanol (50 mL) was degassed with argon for 0.25 h. To this was added 10% palladium on activated charcoal (0.131 g, 0.123 mmol, 5 mol %) and the suspension was degassed with argon for 0.25 h. Triethylamine (1.71 mL, 12.3 mmol) was added and the mixture was degassed with argon for a further 0.05 h. The reaction mixture was degassed with hydrogen for 1 h and then stirred under an atmosphere of hydrogen for a further 21 h. The crude reaction mixture was filtered through a short pad of Celite with ethyl acetate (100 mL) and concentrated *in vacuo*. Purification by flash column chromatography eluting with 2% methanol in dichloromethane gave *tert*-butyl (2*S*,6*R*)-6-acetamido-2-(*tert*-butoxycarbonylamino)-6-phenylhexanoate (**13**) as a white solid (0.979 g, 95%). Mp 97–99 °C; IR (neat) 3376, 3341, 2982, 2931, 1733, 1691, 1648, 1517, 1369, 1353, 1151, 1066, 702 cm^–1^; [α]_D_^18^ +74.6 (*c* 0.1, CHCl_3_); ^1^H NMR (400 MHz, CDCl_3_): δ 7.35–7.28 (m, 2H), 7.26–7.19 (m, 3H), 5.99 (d, 1H, *J* = 7.2 Hz), 5.00 (d, 1H, *J* = 8.0 Hz), 4.94 (dt, 1H, *J* = 8.0, 7.2 Hz), 4.18–4.02 (m, 1H), 1.97 (s, 3H), 1.87–1.78 (m, 2H), 1.77–1.68 (m, 1H), 1.63–1.54 (m, 1H), 1.43 (s, 9H), 1.41 (s, 9H), 1.33–1.23 (m, 2H); ^13^C{^1^H} NMR (101 MHz, CDCl_3_): δ 172.0, 169.3, 155.6, 141.9, 128.8, 127.5, 126.7, 82.0, 79.8, 53.7, 53.4, 35.3, 33.1, 28.5, 28.1, 23.5, 21.8; MS (APCI) *m*/*z* 421 (M + H^+^, 100); HRMS (APCI) *m*/*z*: [M + H]^+^ Calcd for C_23_H_36_N_2_O_5_H 421.2697; Found 421.2699.

### 1-*tert*-Butyl 7-Methyl (2*S*,6*R*)-6-Acetamido-2-(*tert*-butoxycarbonylamino)heptane-1,7-dioate (**15**)

To a stirred solution of sodium periodate (0.642 g, 3.00 mmol) in water (4.8 mL) was added dichloroethane (3.2 mL), acetonitrile (3.2 mL), and *tert*-butyl (2*S*,6*R*)-6-acetamido-2-(*tert*-butoxycarbonylamino)-6-phenylhexanoate (**13**) (0.0841 g, 0.200 mmol), followed by ruthenium(III) chloride hydrate (0.00830 g, 0.0400 mmol). The reaction mixture was vigorously stirred at room temperature for 22 h. Ruthenium(III) chloride hydrate (0.00830 g, 0.0400 mmol) was added and the mixture was vigorously stirred for a further 19 h. The resultant white suspension was diluted with ethyl acetate (60 mL), filtered through a glass sinter funnel, and washed with water (30 mL). The organic layer was dried (MgSO_4_), filtered and concentrated *in vacuo* to afford the carboxylic acid **14** as a dark brown oil which was used without further purification. Carboxylic acid **14** was dissolved in anhydrous toluene (1.2 mL) and anhydrous methanol (0.8 mL) under argon, and then 1.8 M (trimethylsilyl)diazomethane in hexanes solution (0.334 mL, 0.601 mmol) was added dropwise. The reaction mixture was stirred at room temperature for 2 h and concentrated *in vacuo*. Purification by flash column chromatography eluting with dichloromethane and then a gradient of 1–2% methanol in dichloromethane gave 1-*tert*-butyl 7-methyl (2*S*,6*R*)-6-acetamido-2-(*tert*-butoxycarbonylamino)heptan-1,7-dioate (**15**) as a colorless oil (0.0521 g, 65%). IR (neat) 3374, 3345, 2981, 2933, 1734, 1689, 1655, 1517, 1369, 1352, 1221, 1151 cm^–1^; [α]_D_^20^ +12.5 (*c* 0.1, CHCl_3_); ^1^H NMR (400 MHz, CDCl_3_): δ 6.28 (d, 1H, *J* = 6.8 Hz), 5.07 (d, 1H, *J* = 7.2 Hz), 4.59–4.53 (m, 1H), 4.14–4.08 (m, 1H), 3.71 (s, 3H), 2.00 (s, 3H), 1.89–1.51 (m, 4H), 1.46–1.37 (m, 19H), 1.34–1.24 (m, 1H); ^13^C{^1^H} NMR (101 MHz, CDCl_3_): δ 172.9, 171.8, 170.0, 155.6, 82.1, 79.9, 53.6, 52.4, 52.0, 32.9, 31.7, 28.4, 28.1, 23.2, 21.0; MS (ESI) *m*/*z* 403 (M + H^+^, 100); HRMS (ESI) *m*/*z*: [M + H]^+^ Calcd for C_19_H_34_N_2_O_7_H 403.2439; Found 403.2443.

### *meso*-2,6-Diaminopimelic Acid Dihydrochloride (**4**)^21^

To a stirred solution of sodium periodate (1.93 g, 9.00 mmol) in water (15 mL) was added dichloroethane (10 mL), acetonitrile (10 mL), and *tert*-butyl (2*S*,6*R*)-6-acetamido-2-(*tert*-butoxycarbonylamino)-6-phenylhexanoate (**13**) (0.252 g, 0.600 mmol), followed by ruthenium(III) chloride hydrate (0.0249 g, 0.120 mmol). The reaction mixture was vigorously stirred at room temperature for 22 h. Ruthenium(III) chloride hydrate (0.0249 g, 0.120 mmol) was added and the mixture was vigorously stirred for a further 19 h. The resultant white suspension was diluted with ethyl acetate (80 mL), filtered through a glass sinter funnel, and washed with water (40 mL). The organic layer was dried (MgSO_4_), filtered and concentrated *in vacuo*. Purification by flash column chromatography eluting with dichloromethane and then a gradient of 10–50% methanol in dichloromethane, followed by filtration through activated carbon with methanol (20 mL) gave (2*R*,6*S*)-2-acetamido-7-(*tert*-butoxy)-6-(*tert*-butoxycarbonylamino)-7-oxoheptanoic acid (**14**) as a white solid (0.150 g, 65%). (2*R*,6*S*)-2-Acetamido-7-(*tert*-butoxy)-6-(*tert*-butoxycarbonylamino)-7-oxoheptanoic acid (**14**) (0.118 g, 0.303 mmol) was suspended in 6 M aqueous hydrochloric acid solution (5 mL) and stirred under reflux for 4 h. The reaction mixture was cooled to room temperature and concentrated *in vacuo*. The resultant residue was dissolved in methanol (1 mL), warmed to 40 °C, and then diethyl ether (9 mL) was added dropwise, which produced a white precipitate. The suspension was cooled to room temperature, the supernatant was removed and the precipitate was washed with diethyl ether (4 mL). The supernatant was concentrated *in vacuo* and then recrystallized from the minimum amount of hot ethanol to afford a white precipitate. The combined precipitates were dried under high vacuum to give *meso*-2,6-diaminopimelic acid dihydrochloride (**4**) as a white solid (0.0631 g, 79%). Mp > 250 °C (decomposition); lit.^21^ 250–252 °C (decomposition); Spectroscopic data were consistent with the literature.^21^

### *tert*-Butyl (2*S*,4*S*,5*E*)-2-(*tert*-Butoxycarbonylamino)-4-hydroxy-6-phenylhex-5-enoate (**16**) Using Stoichiometric (*R*)-(+)-2-Methyl-CBS-oxazaborolidine

To a solution of *tert*-butyl (2*S*,5*E*)-2-(*tert*-butoxycarbonylamino)-4-oxo-6-phenylhex-5-enoate (**9**) (0.10 g, 0.27 mmol) in THF (10 mL) was added dropwise with stirring (*R*)-(+)-2-methyl-CBS-oxazaborolidine (0.30 mL, 0.30 mmol, 1 M solution in toluene) at 0 °C. The mixture was allowed to stir for 0.5 h at 0 °C. Borane (0.80 mL, 0.80 mmol, 1 M in THF) was added dropwise and stirring was continued at 0 °C for 4 h. The mixture was quenched with methanol (5 mL), warmed to room temperature, and concentrated *in vacuo*. The reaction mixture was dissolved in diethyl ether (30 mL) and washed with 1 M citric acid (3 × 50 mL), water (50 mL), brine (50 mL) and dried (MgSO_4_), filtered and concentrated *in vacuo*. Purification by flash column chromatography on silica eluting with 15% ethyl acetate in petroleum ether gave *tert*-butyl (2*S*,4*S*,5*E*)-2-(*tert*-butoxycarbonylamino)-4-hydroxy-6-phenylhex-5-enoate (**16**) as a white solid (0.092 g, 87%). Mp 120–123 °C; IR (neat) 3457, 2981, 2365, 1723, 1685, 1502, 1362, 1243, 1152, 909, 746 cm^–1^; [α]_D_^25^ +47.0 (*c* 0.1, CHCl_3_); ^1^H NMR (400 MHz, CDCl_3_): δ 7.40–7.35 (m, 2H), 7.32–7.27 (m, 2H), 7.25–7.18 (m, 1H), 6.66 (d, *J* = 15.9 Hz, 1H), 6.23 (dd, *J* = 15.9, 8.1 Hz, 1H), 5.49 (d, *J* = 7.8 Hz, 1H), 4.44 (ddd, *J* = 10.7, 8.1, 4.0 Hz, 1H), 4.39–4.29 (m, 2H), 2.03 (ddd, *J* = 13.9, 10.7, 4.0 Hz, 1H), 1.71–1.63 (m, 1H), 1.46 (s, 18H); ^13^C{^1^H} NMR (101 MHz, CDCl_3_): δ 171.9, 157.1, 137.0, 131.1, 129.7, 128.7, 127.6, 126.6, 82.6, 80.8, 68.1, 51.3, 42.3, 28.4, 28.1; MS (ESI) *m*/*z* 400 (M + Na^+^, 100); HRMS (ESI) *m*/*z*: [M + Na]^+^ Calcd for C_21_H_31_NO_5_Na 400.2094; Found 400.2097.

### *tert*-Butyl (2*S*,4*S*,5*E*)-2-(*tert*-Butoxycarbonylamino)-4-hydroxy-6-phenylhex-5-enoate (**16**) Using Catalytic (*R*)-(+)-2-Methyl-CBS-oxazaborolidine

To a stirred solution of (*R*)-(+)-2-methyl-CBS-oxazaborolidine (11.1 mg, 0.0400 mmol) in anhydrous THF (0.8 mL) in an oven-dried flask under argon was added 1 M borane in THF solution (0.400 mL, 0.400 mmol) dropwise at 0 °C. The mixture was stirred for 0.25 h. A solution of *tert*-butyl (2*S*,5*E*)-2-(*tert*-butoxycarbonylamino)-4-oxo-6-phenylhex-5-enoate (**9**) (0.150 g, 0.400 mmol) in anhydrous THF (0.8 mL) was then added dropwise at 0 °C and the reaction mixture was stirred for 4 h. The reaction mixture was quenched with methanol (0.5 mL), allowed to warm to room temperature and concentrated *in vacuo*. Purification by flash column chromatography eluting with 15% ethyl acetate in hexane gave *tert*-butyl (2*S*,4*S*,5*E*)-2-(*tert*-butoxycarbonylamino)-4-hydroxy-6-phenylhex-5-enoate (**16**) as a white solid (0.106 g, 70%). Spectroscopic data as described above.

### *tert*-Butyl (2*S*,4*E*,6*S*)-2-(*tert*-Butoxycarbonylamino)-6-phenyl-6-(trichloromethylcarbonylamino)hex-4-enoate (**18**)

The reaction was carried out according to the previously described procedure for **12** using *tert*-butyl (2*S*,4*S*,5*E*)-2-(*tert*-butoxycarbonylamino)-4-hydroxy-6-phenylhex-5-enoate (**16**) (0.377 g, 1.00 mmol), anhydrous dichloromethane (14 mL), trichloroacetonitrile (0.150 mL, 1.50 mmol), 1,8-diazabicyclo[5.4.0]undec-7-ene (0.0750 mL, 0.500 mmol), anhydrous *p*-xylene (4.55 mL) and anhydrous potassium carbonate (0.0137 g, 0.0991 mmol). Purification by flash column chromatography eluting with 15% ethyl acetate in hexane gave *tert*-butyl (2*S*,4*E*,6*S*)-2-(*tert*-butoxycarbonylamino)-6-phenyl-6-(trichloromethylcarbonylamino)hex-4-enoate (**18**) as a colorless oil (0.346 g, 66%). IR (neat) 3328, 2905, 1700, 1506, 1362, 1217, 1152, 913, 768 cm^–1^; [α]_D_^25^ +31.0 (*c* 0.1, CHCl_3_); ^1^H NMR (400 MHz, CDCl_3_): δ 7.41–7.29 (m, 5H), 6.89 (d, *J* = 7.6 Hz, 1H), 5.77 (dd, *J* = 15.4, 5.8 Hz, 1H), 5.62 (dt, *J* = 15.4, 6.5 Hz, 1H), 5.55–5.49 (m, 1H), 5.10 (d, *J* = 8.0 Hz, 1H), 4.31–4.24 (m, 1H), 2.63 (dt, *J* = 13.8, 6.5 Hz, 1H), 2.51 (dt, *J* = 13.8, 6.5 Hz, 1H), 1.42 (s, 18H); ^13^C{^1^H} NMR (101 MHz, CDCl_3_): δ 170.9, 160.9, 155.2, 139.1, 131.8, 129.2, 128.4, 127.9, 127.1, 92.7, 82.4, 79.9, 56.7, 53.5, 35.5, 28.5, 28.1; MS (ESI) *m*/*z* 543 (M + Na^+^, 100); HRMS (ESI) *m*/*z*: [M + Na]^+^ Calcd for C_23_H_31_^35^Cl_3_N_2_O_5_Na 543.1191; Found 543.1194.

### *tert*-Butyl (2*S*,6*S*)-6-Acetamido-2-(*tert*-butoxycarbonylamino)-6-phenylhexanoate (**19**)

The reaction was carried out according to the previously described procedure for **13** using *tert*-butyl (2*S*,4*E*,6*S*)-2-(*tert*-butoxycarbonylamino)-6-phenyl-6-(trichloromethylcarbonylamino)hex-4-enoate (**18**) (0.0783 g, 0.150 mmol), methanol (3 mL), and 10% palladium on activated charcoal (0.00800 g, 0.00750 mmol, 5 mol %). The solution was degassed with argon for a total of 10 min. Triethylamine (0.105 mL, 0.750 mmol) was added and the mixture was degassed with argon for a further 1 min. The reaction mixture was degassed with hydrogen for 10 min and then stirred under an atmosphere of hydrogen for a further 22 h. Purification by flash column chromatography eluting with 5% methanol in dichloromethane gave *tert*-butyl (2*S*,6*S*)-6-acetamido-2-(*tert*-butoxycarbonylamino)-6-phenylhexanoate (**19**) as a white solid (0.0598 g, 95%). Mp 162–164 °C; IR (neat) 3341, 3254, 2978, 1732, 1694, 1656, 1535, 1366, 1287, 1150, 702 cm^–1^; [α]_D_^17^ −14.5 (*c* 0.1, CHCl_3_); ^1^H NMR (400 MHz, CDCl_3_): δ 7.35–7.20 (m, 5H), 5.89 (br s, 1H), 5.08 (d, 1H, *J* = 7.2 Hz), 4.91 (dt, 1H, *J* = 7.6, 7.2 Hz), 4.16–4.09 (m, 1H), 1.98 (s, 3H), 1.92–1.54 (m, 4H), 1.51–1.36 (m, 19H), 1.33–1.23 (m, 1H); ^13^C{^1^H} NMR (101 MHz, CDCl_3_): δ 172.0, 169.5, 155.8, 142.6, 128.8, 127.5, 126.6, 82.0, 79.8, 53.5, 53.4, 35.8, 33.1, 28.5, 28.1, 23.6, 22.1; MS (APCI) *m*/*z* 421 (M + H^+^, 100); HRMS (APCI) *m*/*z*: [M + H]^+^ Calcd for C_23_H_36_N_2_O_5_H 421.2697; Found 421.2701.

### 1-*tert*-Butyl 7-Methyl (2*S*,6*S*)-6-Acetamido-2-(*tert*-butoxycarbonylamino)heptane-1,7-dioate (**20**)

The reaction was carried out according to the previously described procedure for **15**, using sodium periodate (321 mg, 1.50 mmol), water (2.4 mL), dichloroethane (1.6 mL), acetonitrile (1.6 mL), *tert*-butyl (2*S*,6*S*)-6-acetamido-2-(*tert*-butoxycarbonylamino)-6-phenylhexanoate (**19**) (0.0420 g, 0.100 mmol), and ruthenium(III) chloride hydrate (0.00410 g, 0.0200 mmol). The reaction mixture was vigorously stirred at room temperature for 24 h. Ruthenium(III) chloride hydrate (0.00410 g, 0.0200 mmol) was added and the mixture was vigorously stirred for a further 20 h. The subsequent reaction was carried out using anhydrous toluene (0.6 mL), anhydrous methanol (0.4 mL), and 1.8 M (trimethylsilyl)diazomethane in hexanes solution (0.167 mL, 0.301 mmol). The reaction mixture was stirred at room temperature for 2 h and concentrated *in vacuo*. Purification by flash column chromatography eluting with 80% ethyl acetate in hexane gave 1-*tert*-butyl 7-methyl (2*S*,6*S*)-6-acetamido-2-(*tert*-butoxycarbonylamino)heptan-1,7-dioate (**20**) as a colorless oil (0.0255 g, 63%). IR (neat) 3326, 2977, 2931, 1712, 1660, 1523, 1366, 1250, 1150, 1051, 1020, 847 cm^–1^; [α]_D_^18^ +11.2 (*c* 0.1, CHCl_3_); ^1^H NMR (400 MHz, CDCl_3_): δ 6.18 (d, 1H, *J* = 6.8 Hz), 5.10 (d, 1H, *J* = 7.2 Hz), 4.67–4.43 (m, 1H), 4.15–4.08 (m, 1H), 3.73 (s, 3H), 2.01 (s, 3H), 1.88–1.70 (m, 3H), 1.66–1.55 (m, 1H), 1.49–1.35 (m, 20H); ^13^C{^1^H} NMR (101 MHz, CDCl_3_): δ 173.1, 171.9, 170.1, 155.8, 82.1, 79.8, 53.4, 52.5, 52.2, 32.9, 31.8, 28.5, 28.1, 23.3, 21.3; MS (ESI) *m*/*z* 403 (M + H^+^, 100); HRMS (ESI) *m*/*z*: [M + H]^+^ Calcd for C_19_H_34_N_2_O_7_H 403.2439; Found 403.2439.

### (2*S*,6*S*)-2-Acetamido-7-(*tert*-butoxy)-6-(*tert*-butoxycarbonylamino)-7-oxoheptanoic Acid (**21**)

To a stirred solution of sodium periodate (0.963 g, 4.50 mmol) in water (7.5 mL) was added dichloroethane (5 mL), acetonitrile (5 mL), and *tert*-butyl (2*S*,6*S*)-6-acetamido-2-(*tert*-butoxycarbonylamino)-6-phenylhexanoate (**19**) (0.126 g, 0.300 mmol), followed by ruthenium(III) chloride hydrate (0.0124 g, 0.0600 mmol). The reaction mixture was vigorously stirred at room temperature for 24 h. Ruthenium(III) chloride hydrate (0.0124 g, 0.0600 mmol) was added and the mixture was vigorously stirred for a further 18 h. The resultant white suspension was diluted with ethyl acetate (80 mL), filtered through a glass sinter funnel, and washed with water (40 mL). The organic layer was dried (MgSO_4_), filtered and concentrated *in vacuo*. Purification by flash column chromatography eluting with dichloromethane and then a gradient of 10–50% methanol in dichloromethane gave (2*S*,6*S*)-2-acetamido-7-(*tert*-butoxy)-6-(*tert*-butoxycarbonylamino)-7-oxoheptanoic acid (**21**) as a white solid (0.0853 g, 73%). Mp 124–126 °C; IR (neat) 3297, 2979, 2933, 1702, 1584, 1391, 1365, 1250, 1150, 1045, 1018, 845 cm^–1^; [α]_D_^17^ +10.4 (*c* 0.1, CH_3_OH); ^1^H NMR (400 MHz, CD_3_OD): δ 4.26–4.20 (m, 1H), 3.98–3.81 (m, 1H), 1.99 (s, 3H), 1.89–1.79 (m, 1H), 1.76–1.58 (m, 3H), 1.55–1.36 (m, 20H); ^13^C{^1^H} NMR (101 MHz, CD_3_OD): δ 179.7, 173.8, 172.9, 158.1, 82.5, 80.4, 55.9, 55.8, 33.2, 32.5, 28.8, 28.3, 23.5, 22.8; MS (ESI) *m*/*z* 289 [(M – CO_2_^*t*^Bu) + H^+^, 100]; HRMS (ESI) *m*/*z*: [(M – CO_2_^*t*^Bu) + H]^+^ Calcd for C_13_H_24_N_2_O_5_H 289.1758; Found 289.1769.

### l,l-Diaminopimelic Acid Dihydrochloride (**3**)^23^

(2*S*,6*S*)-2-Acetamido-7-(*tert*-butoxy)-6-(*tert*-butoxycarbonylamino)-7-oxoheptanoic acid (**21**) (0.0544 g, 0.140 mmol) was suspended in 6 M aqueous hydrochloric acid solution (2.5 mL) and stirred under reflux for 4 h. The reaction mixture was cooled to room temperature and concentrated *in vacuo*. The resultant residue was dissolved in methanol (0.5 mL), warmed to 40 °C, and then diethyl ether (4.5 mL) was added dropwise, which produced a white precipitate. The suspension was cooled to room temperature and the supernatant was removed. The precipitate was washed with diethyl ether (2 mL) and dried under high vacuum to give l,l-diaminopimelic acid dihydrochloride (**3**) as a white solid (0.0249 g, 68%). [α]_D_^22^ +38.0 (*c* 0.7, 1 M HCl), lit.^23^ +37.6 (*c* 0.96, 1 M HCl); Spectroscopic data were consistent with the literature.^23^

### 1-*tert*-Butyl (1′*S*,2*S*,6*S*)-6-Acetamido-2-(*tert*-butoxycarbonylamino)-7-[2′-(*tert*-butoxy)-1′-(methyl-2′-oxoethyl)amino]-7-oxoheptanoate (**22**)

To a stirred solution of (2*S*,6*S*)-2-acetamido-7-(*tert*-butoxy)-6-(*tert*-butoxycarbonylamino)-7-oxoheptanoic acid (**21**) (0.0400 g, 0.103 mmol), l-alanine *tert*-butyl ester hydrochloride (0.0205 g, 0.113 mmol), and 1-hydroxybenzotriazole hydrate (0.00700 g, 0.0515 mmol) in acetonitrile (3 mL) at 0 °C was added benzotriazole-1-yl-oxy-tris-pyrrolidinophosphonium hexafluorophosphate (0.0807 g, 0.155 mmol) and diisopropylethylamine (0.0540 mL, 0.309 mmol). The reaction mixture was warmed to room temperature, stirred for 2 h, and concentrated *in vacuo*. The resultant residue was dissolved in ethyl acetate (20 mL) and washed with 1 M aqueous hydrochloric acid solution (20 mL) and then brine (20 mL). The organic layer was dried (MgSO_4_), filtered, and concentrated *in vacuo*. Purification by flash column chromatography eluting with 20% acetonitrile in dichloromethane and then 4% methanol in dichloromethane gave 1-*tert*-butyl (1′*S*,2*S*,6*S*)-6-acetamido-2-(*tert*-butoxycarbonylamino)-7-[2′-(*tert*-butoxy)-1′-(methyl-2′-oxoethyl)amino]-7-oxoheptanoate (**22**) as a white solid (0.0435 g, 82%). Mp 53–55 °C; IR (neat) 3283, 3072, 2979, 2934, 1713, 1646, 1523, 1365, 1249, 1148, 1048, 848 cm^–1^; [α]_D_^20^ +8.8 (*c* 0.1, CHCl_3_); NMR spectra showed a 5:1 mixture of rotational isomers. Data for the major rotamer are reported: ^1^H NMR (400 MHz, CDCl_3_): δ 6.75 (d, 1H, *J* = 7.2 Hz), 6.44 (d, 1H, *J* = 7.6 Hz), 5.19 (d, 1H, *J* = 7.6 Hz), 4.43–4.21 (m, 2H), 4.20–4.00 (m, 1H), 1.99 (s, 3H), 1.86–1.56 (m, 4H), 1.50–1.37 (m, 29H), 1.34 (d, 3H, *J* = 7.2 Hz); ^13^C{^1^H} NMR (101 MHz, CDCl_3_): δ 172.0, 171.9, 171.3, 170.5, 155.8, 82.07, 81.96, 79.7, 53.5, 53.0, 48.9, 32.8, 31.9, 28.5, 28.09, 28.07, 23.2, 21.4, 18.4; MS (ESI) *m*/*z* 516 (M + H^+^, 100); HRMS (ESI) *m*/*z*: [M + H]^+^ Calcd for C_25_H_45_N_3_O_8_H 516.3279; Found 516.3286.

## Data Availability

The data underlying this study are available in the published article and its Supporting Information.

## Supporting Information Available

^1^H and ^13^C NMR spectra of all compounds (PDF)
