# Supplementary material for: Stereoselective Synthesis of meso- and l,l-Diaminopimelic Acids from Enone-Derived α-Amino Acids
Source: J Org Chem. 2024 Jul 2;89(14):10363–70. doi: 10.1021/acs.joc.4c00916 (PMC11267594; doi:10.1021/acs.joc.4c00916)

Supporting Information for:

Stereoselective Synthesis of *meso*- and L,L-Diaminopimelic Acid from Enone-Derived α-Amino Acids

Sineenard Songsri, Holly McErlain and Andrew Sutherland*

School of Chemistry, The Joseph Black Building, University of Glasgow, Glasgow G12 8QQ, UK. Email: Andrew.Sutherland@glasgow.ac.uk.

Table of Contents

1. ^{1}H and ^{13}C NMR Spectra of all Compounds S2–S29

1. ^{1}H and ^{13}C NMR Spectra of all Compounds

^{1}H NMR (400 MHz, $CDCl_3$)

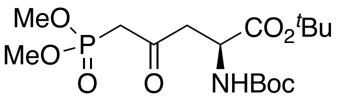

8

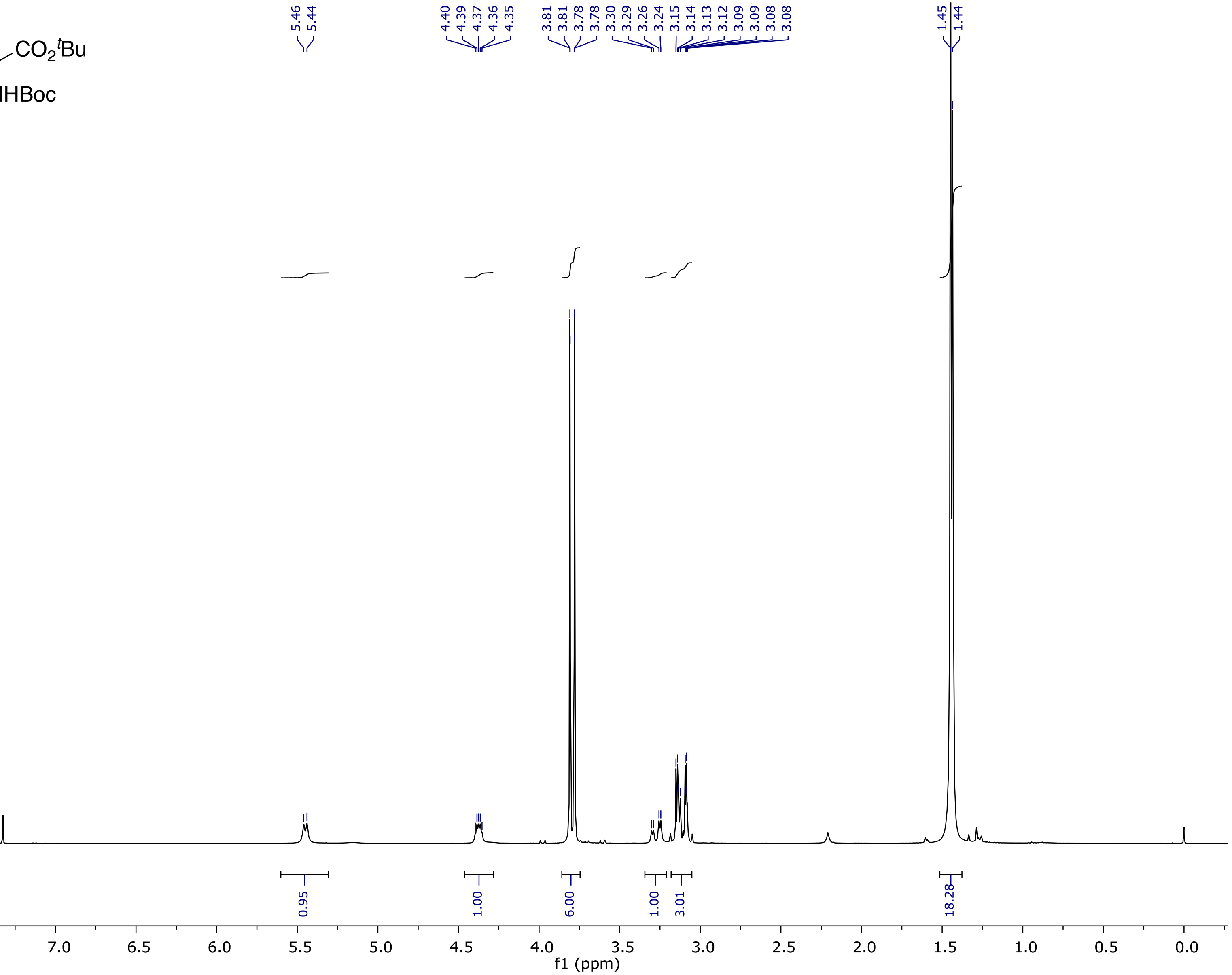

$^{13}C\{^1H\}$ NMR (101 MHz, $CDCl_3$)

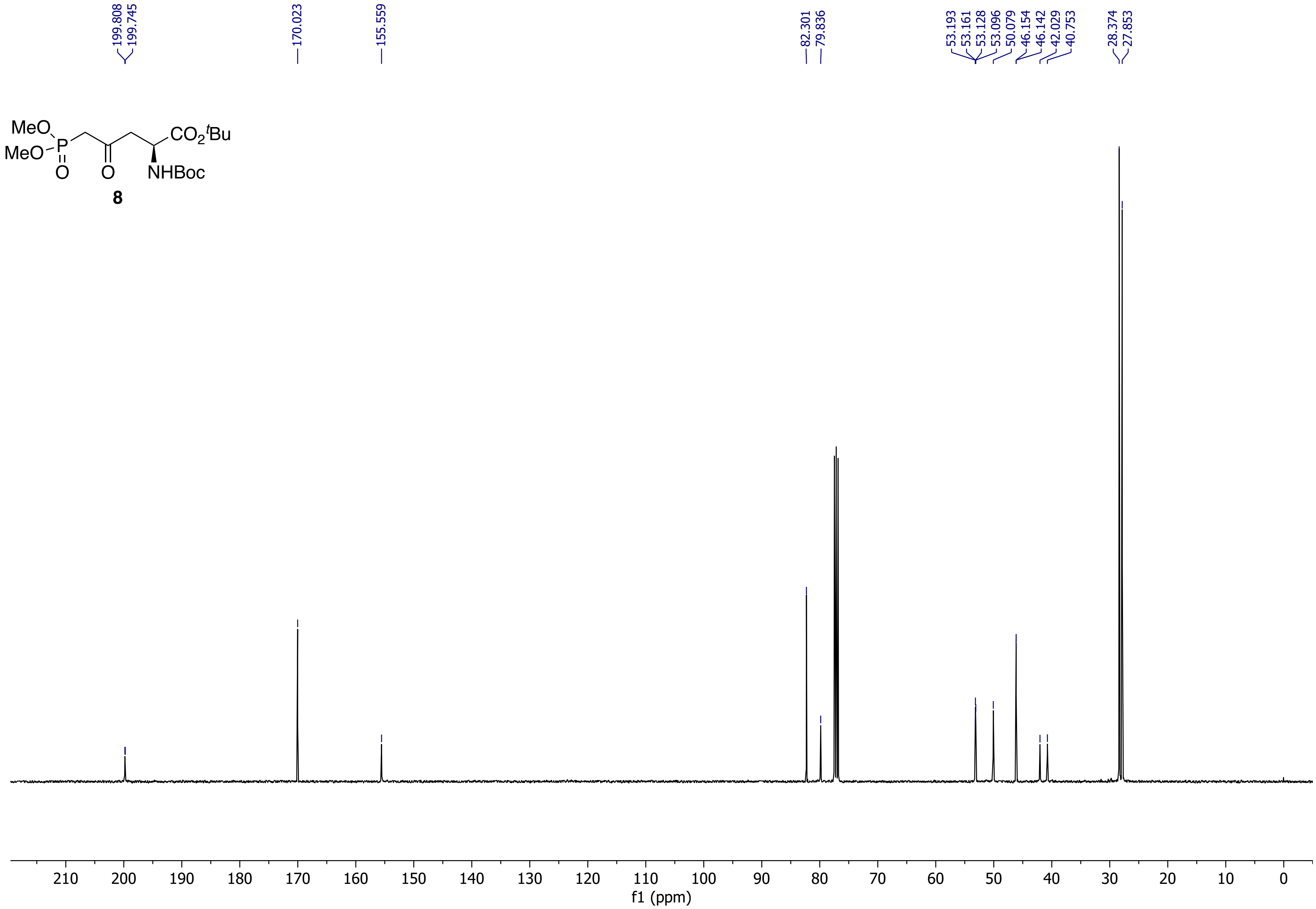

^{1}H NMR (400 MHz, $CDCl_3$)

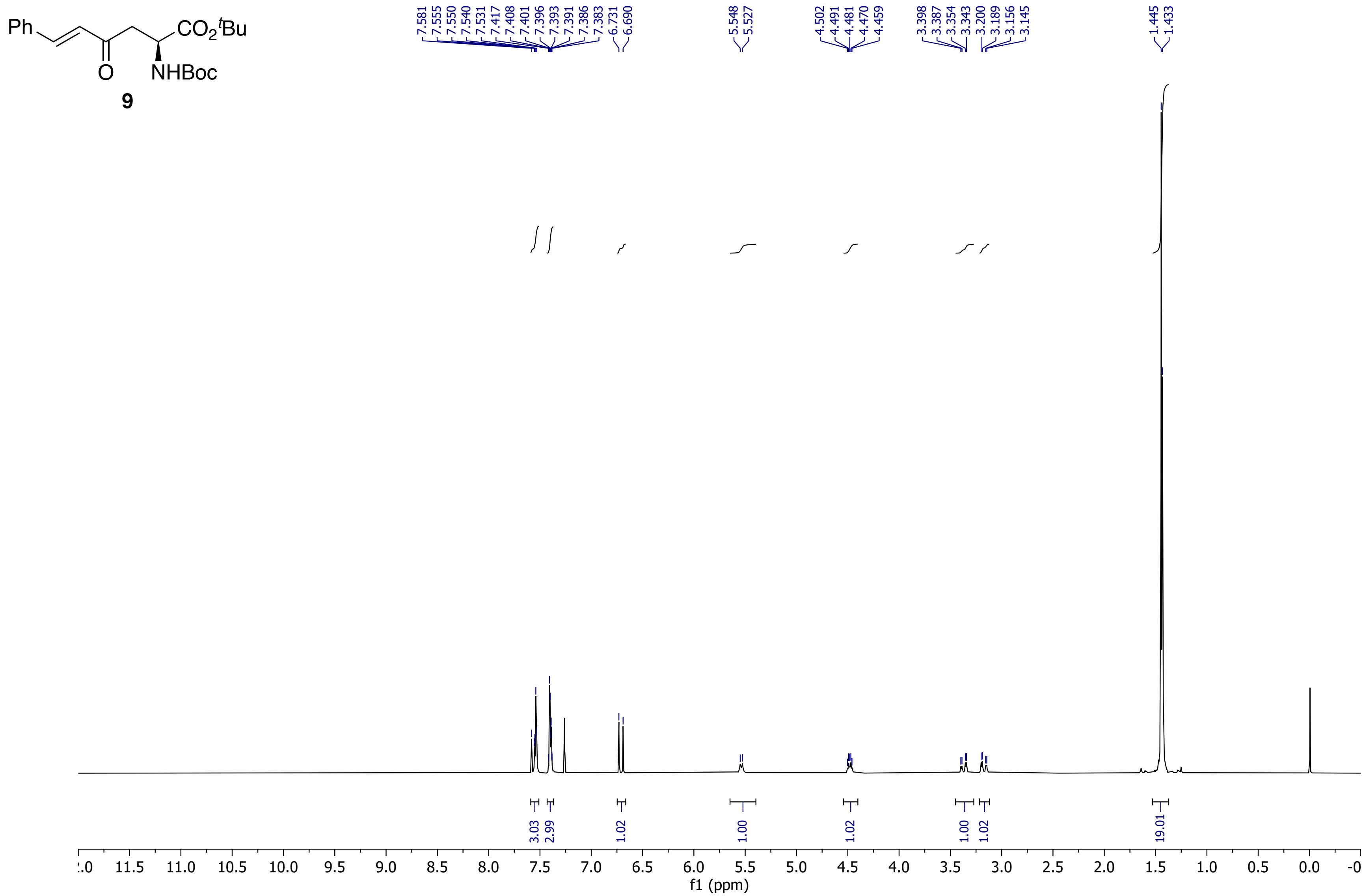

$^{13}C\{^1H\}$ NMR (101 MHz, $CDCl_3$)

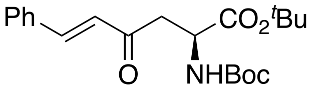

9

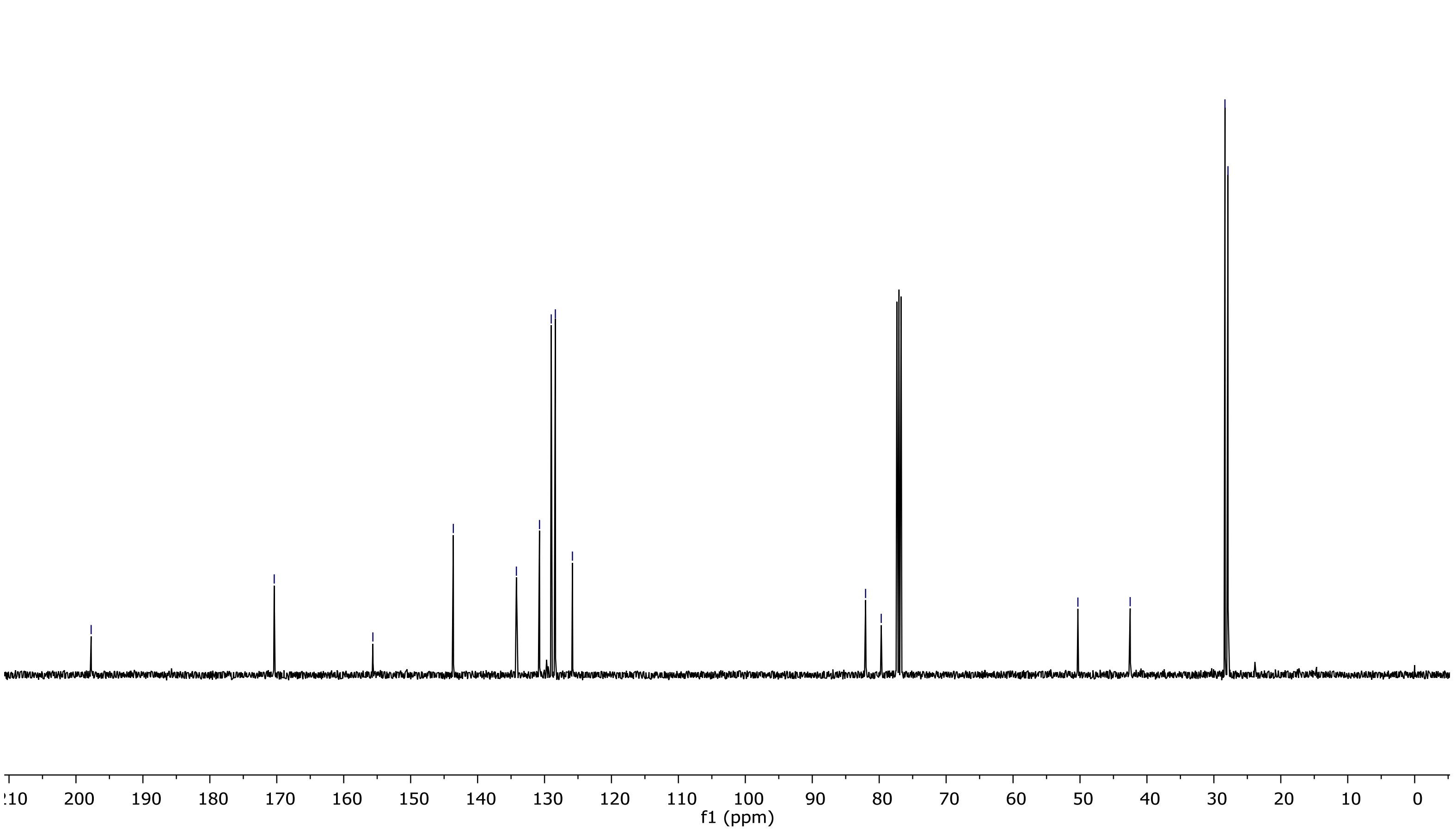

^{1}H NMR (400 MHz, $CDCl_3$)

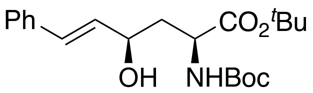

10

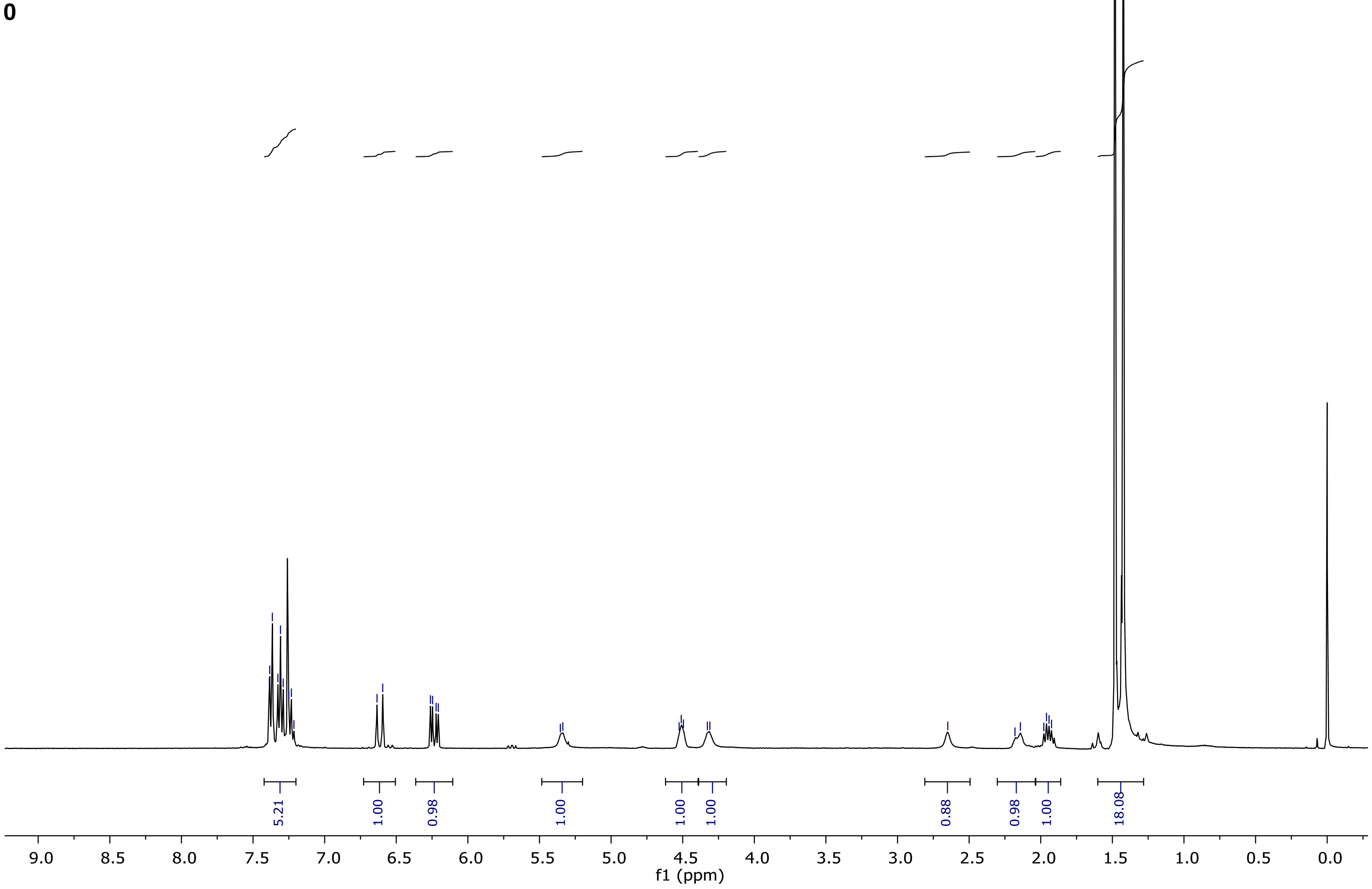

$^{13}C\{^1H\}$ **NMR (101 MHz, $CDCl_3$)**

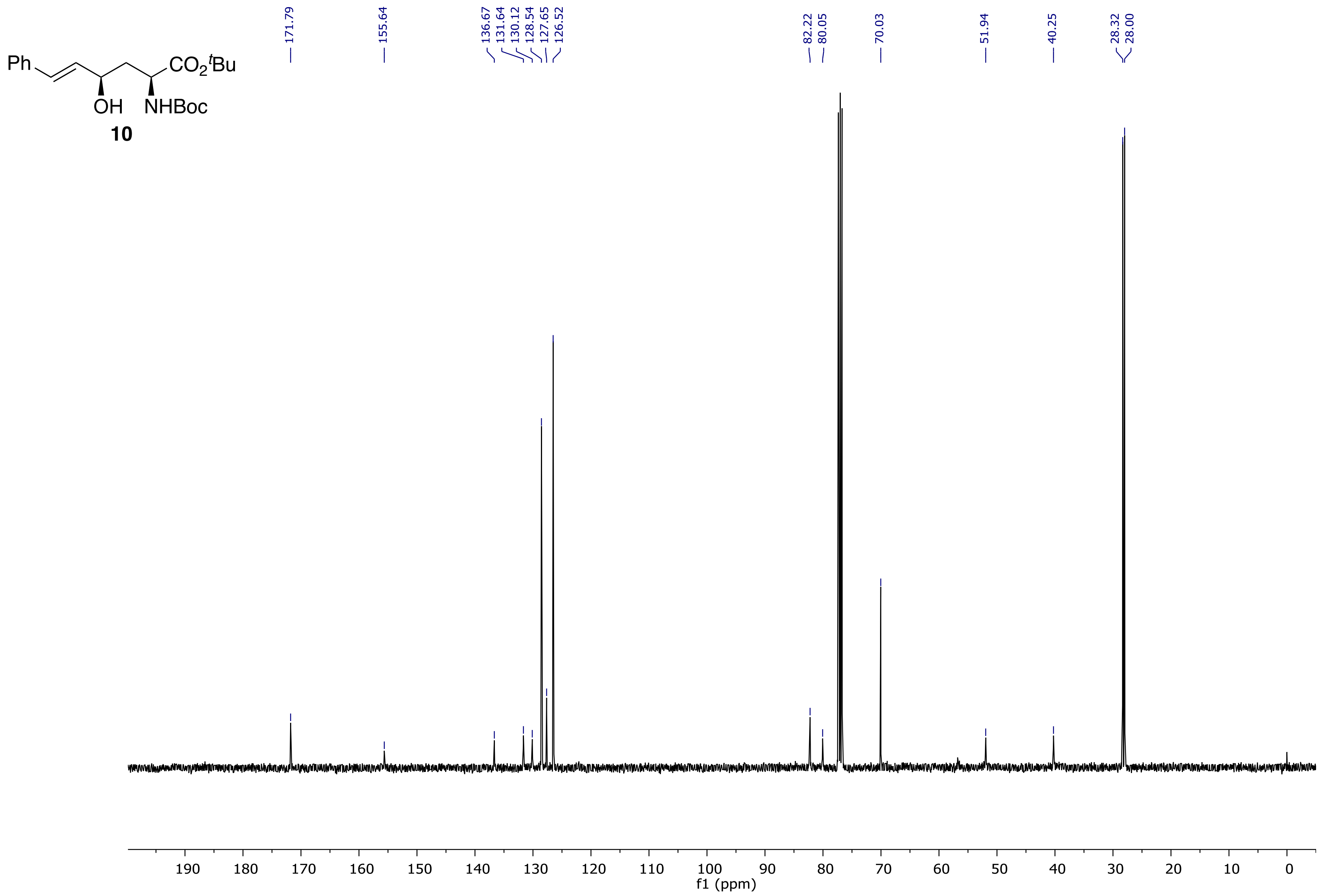

^{1}H NMR (400 MHz, $CDCl_3$)

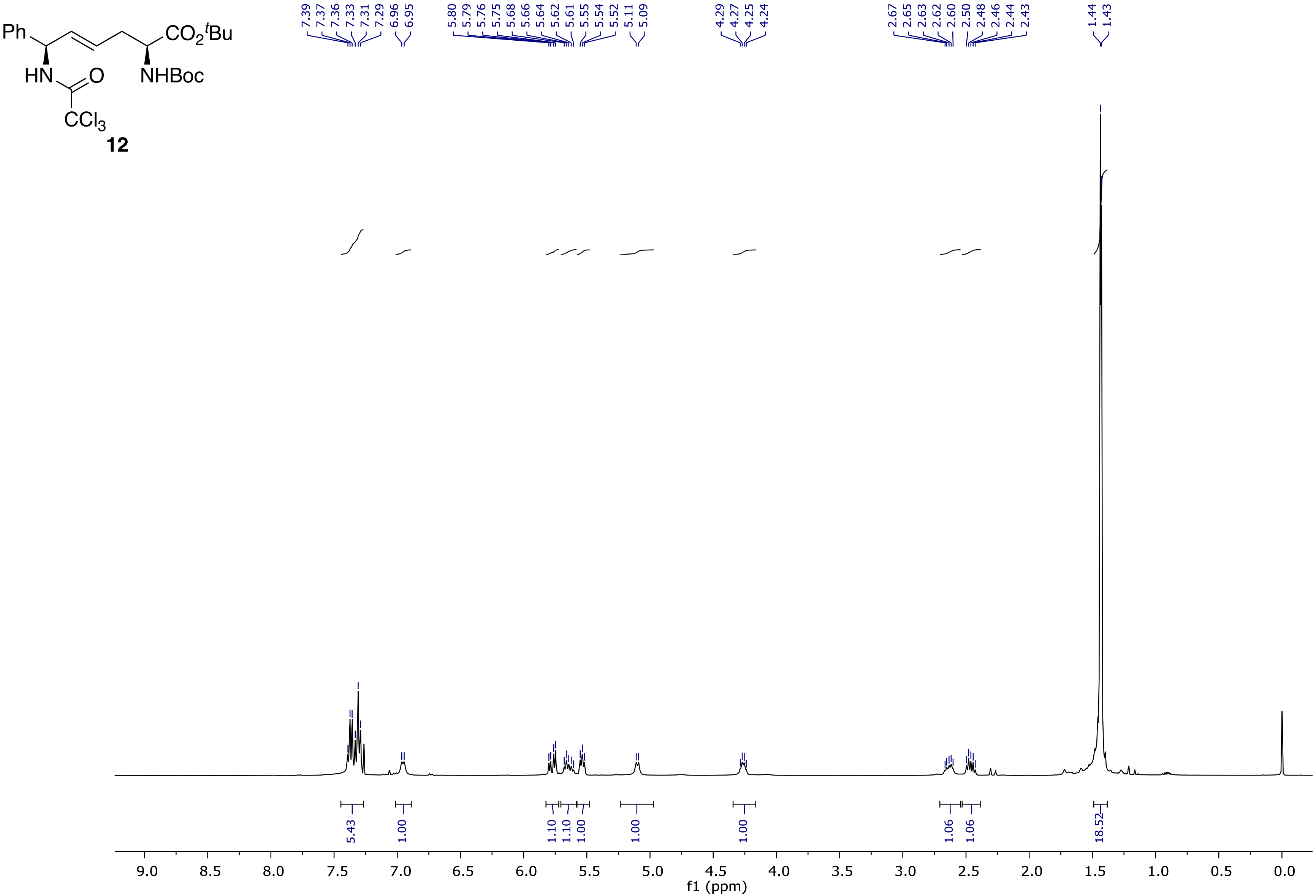

12

$^{13}C\{^1H\}$ NMR (101 MHz, $CDCl_3$)

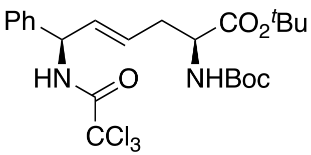

12

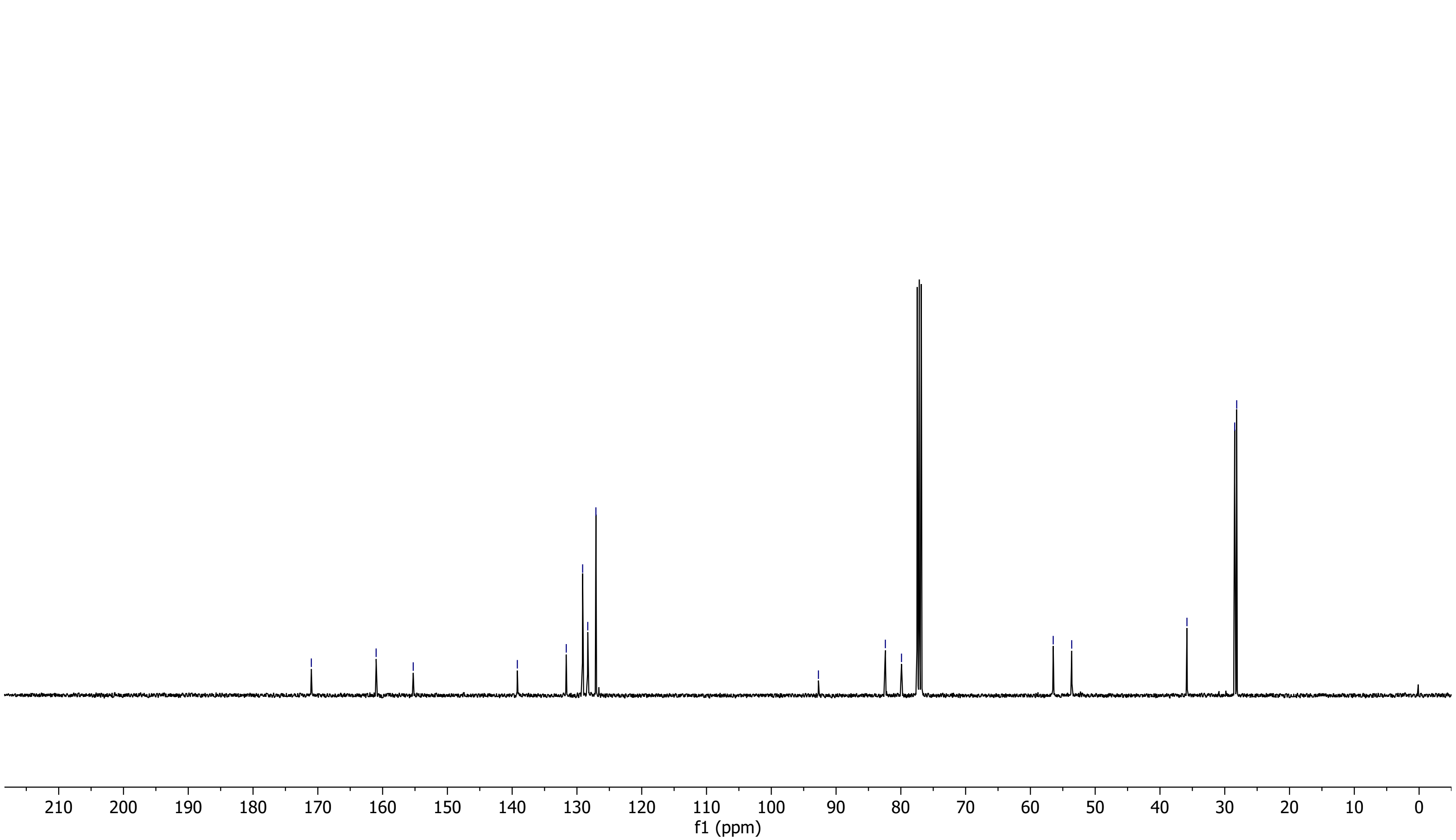

^{1}H NMR (400 MHz, $CDCl_3$)

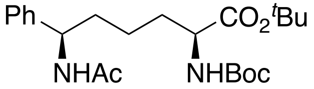

13

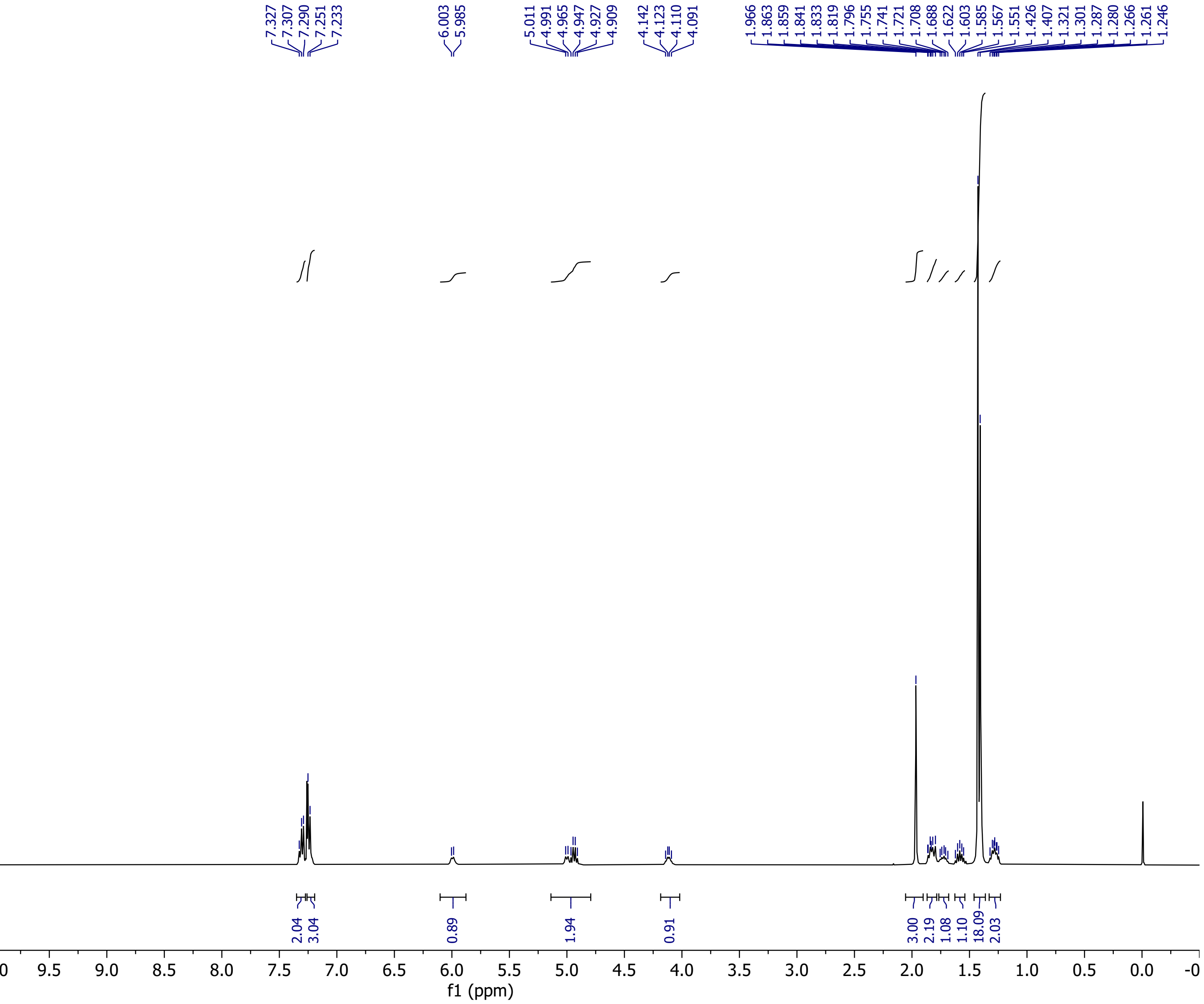

$^{13}C\{^1H\}$ NMR (101 MHz, $CDCl_3$)

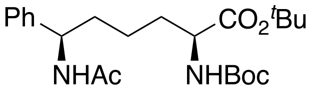

13

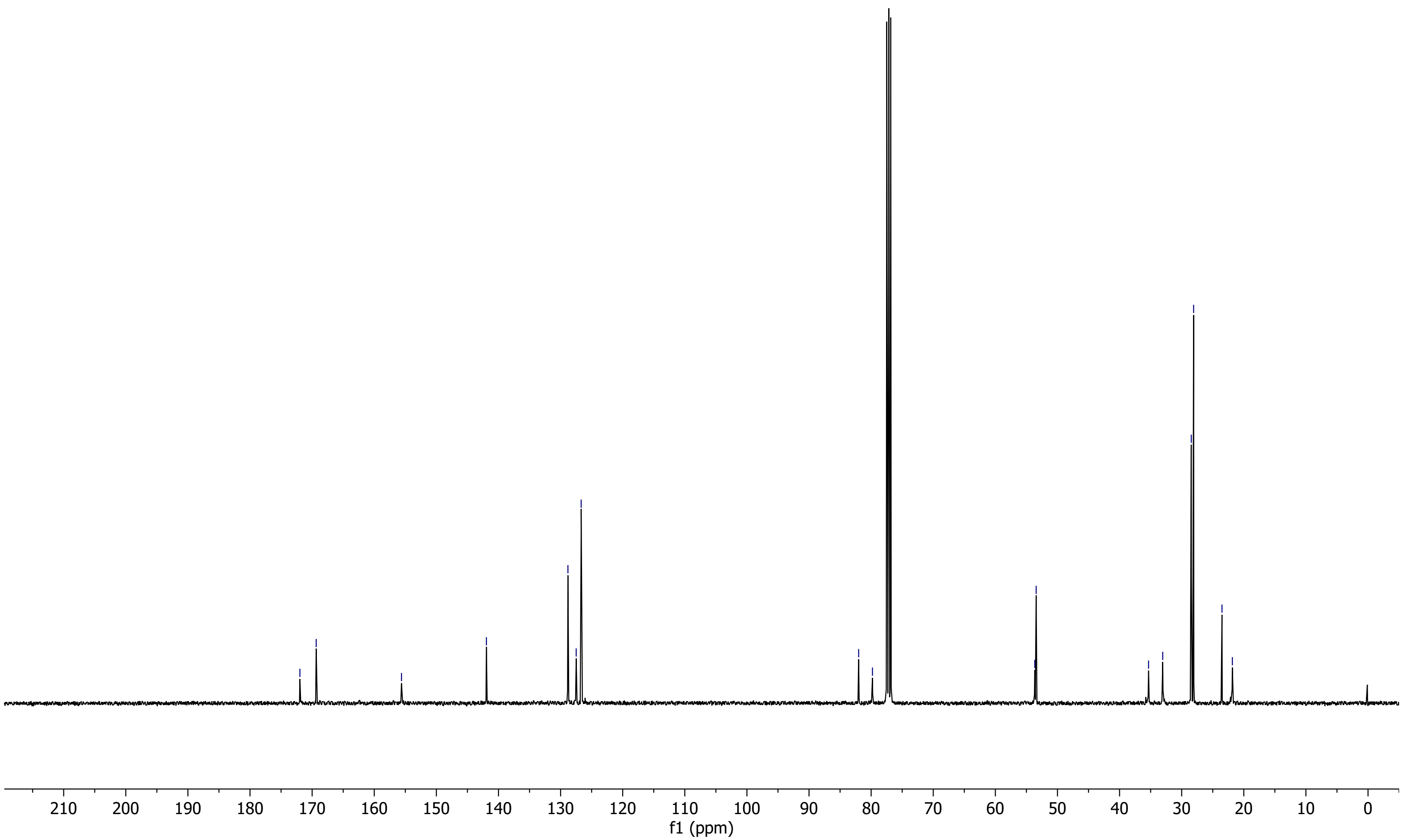

^{1}H NMR (400 MHz, $CDCl_3$)

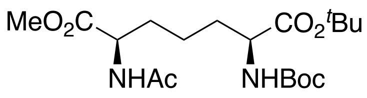

15

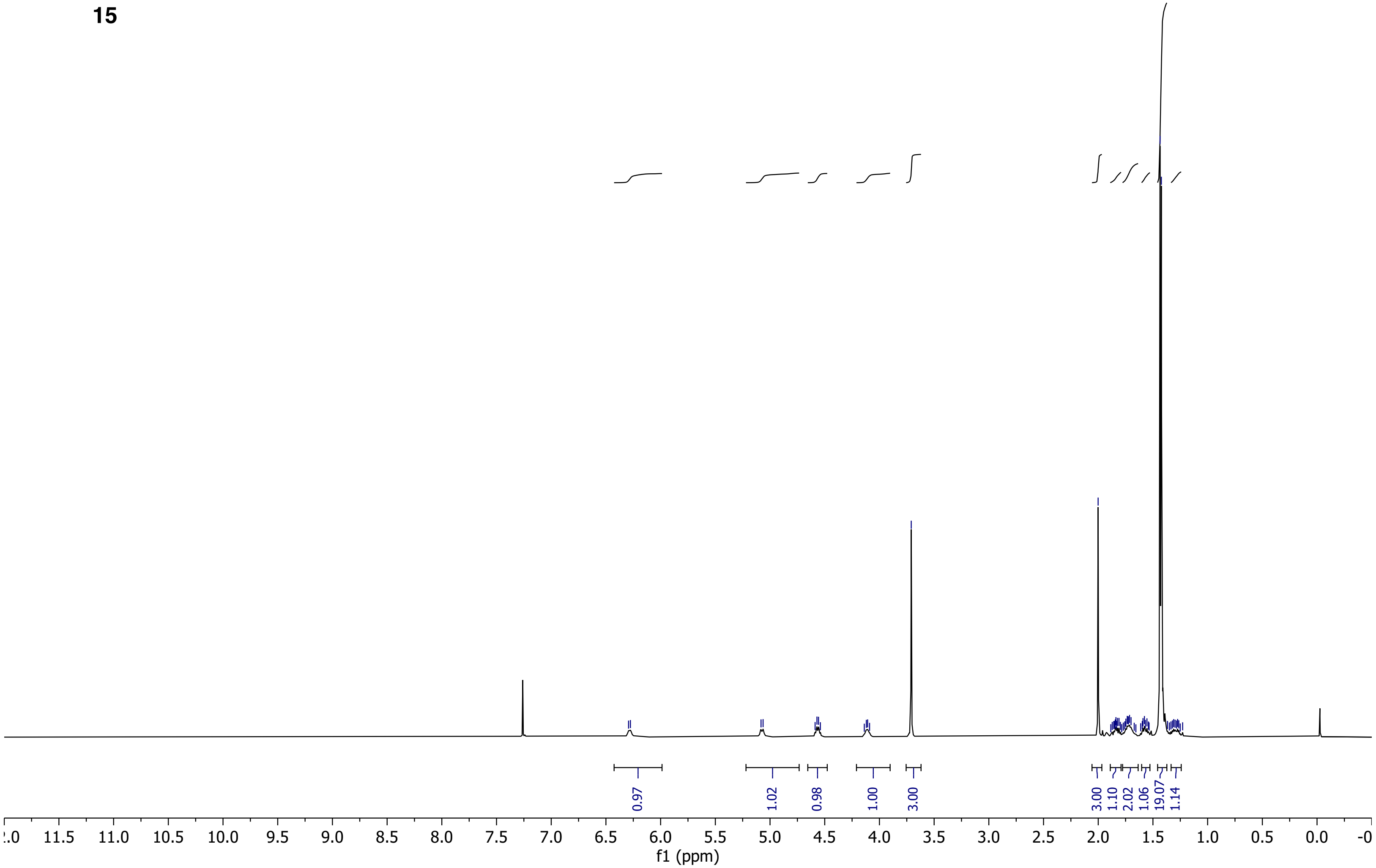

$^{13}C\{^{1}H\}$ NMR (101 MHz, $CDCl_3$)

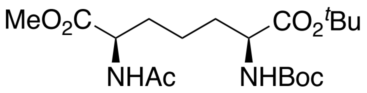

15

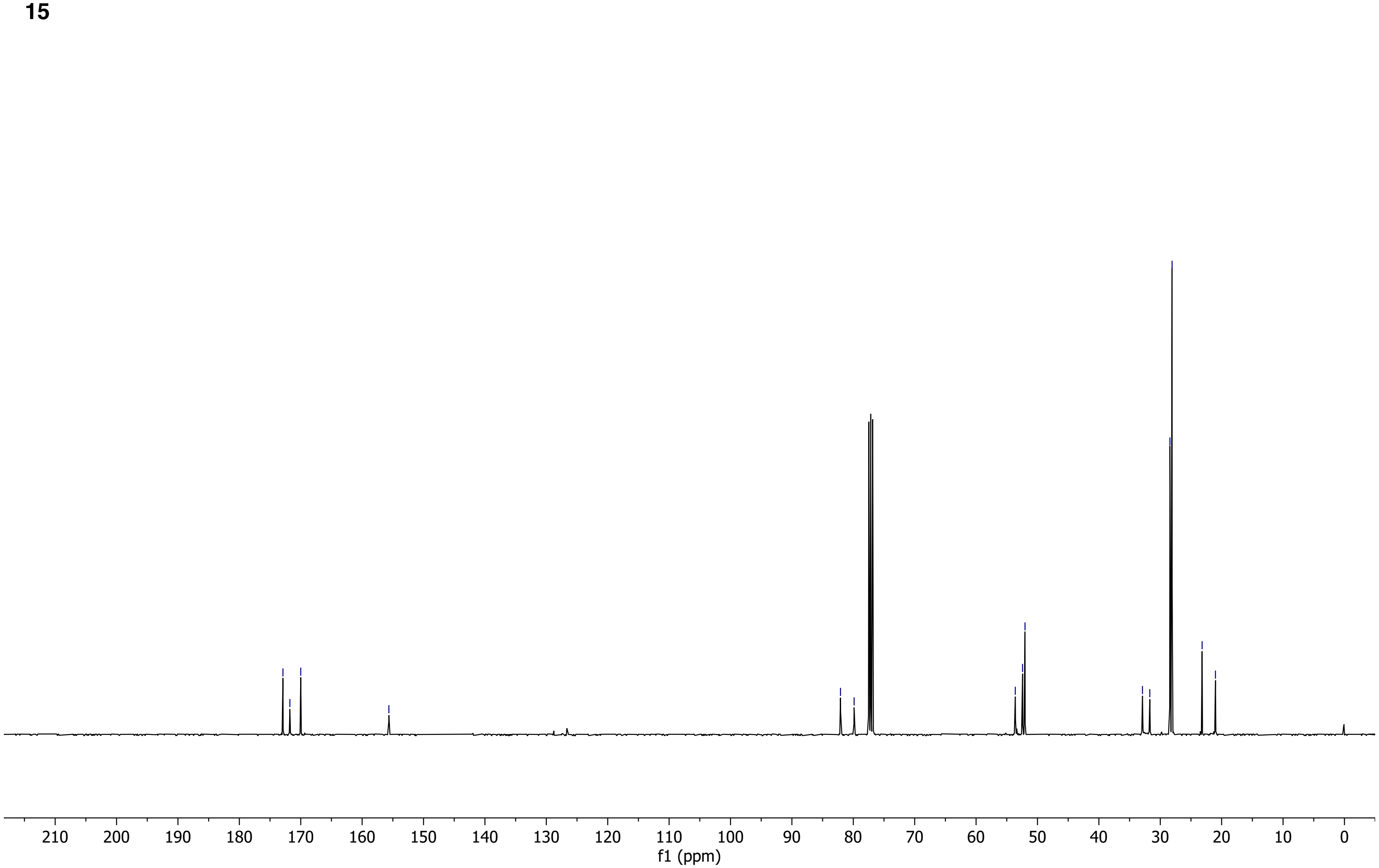

^{1}H NMR (400 MHz, D_2O)

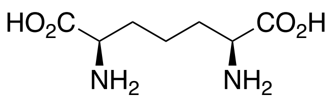

4

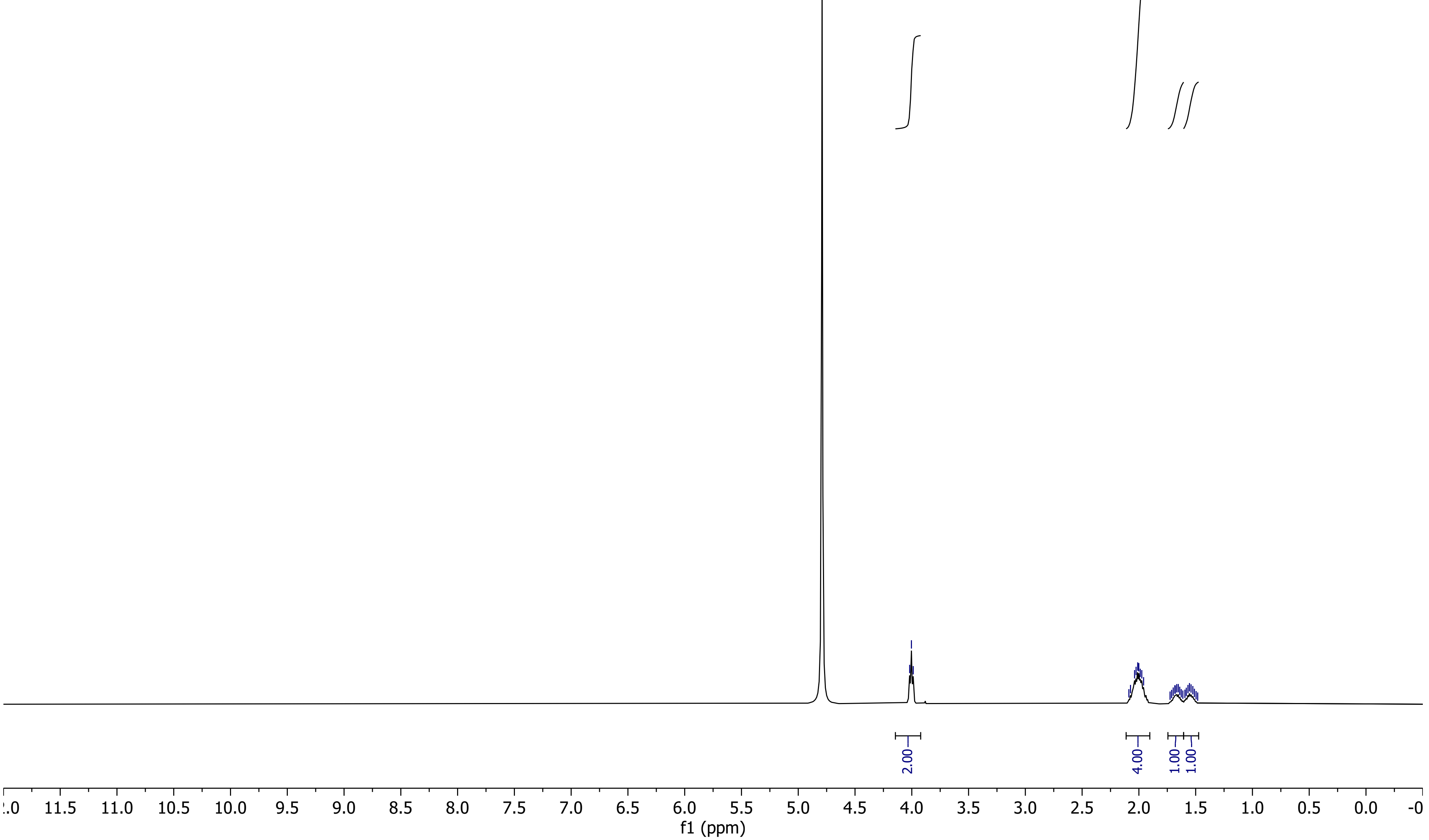

$^{13}C\{^1H\}$ NMR (101 MHz, D_2O)

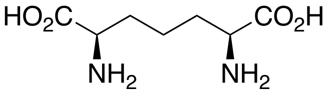

4

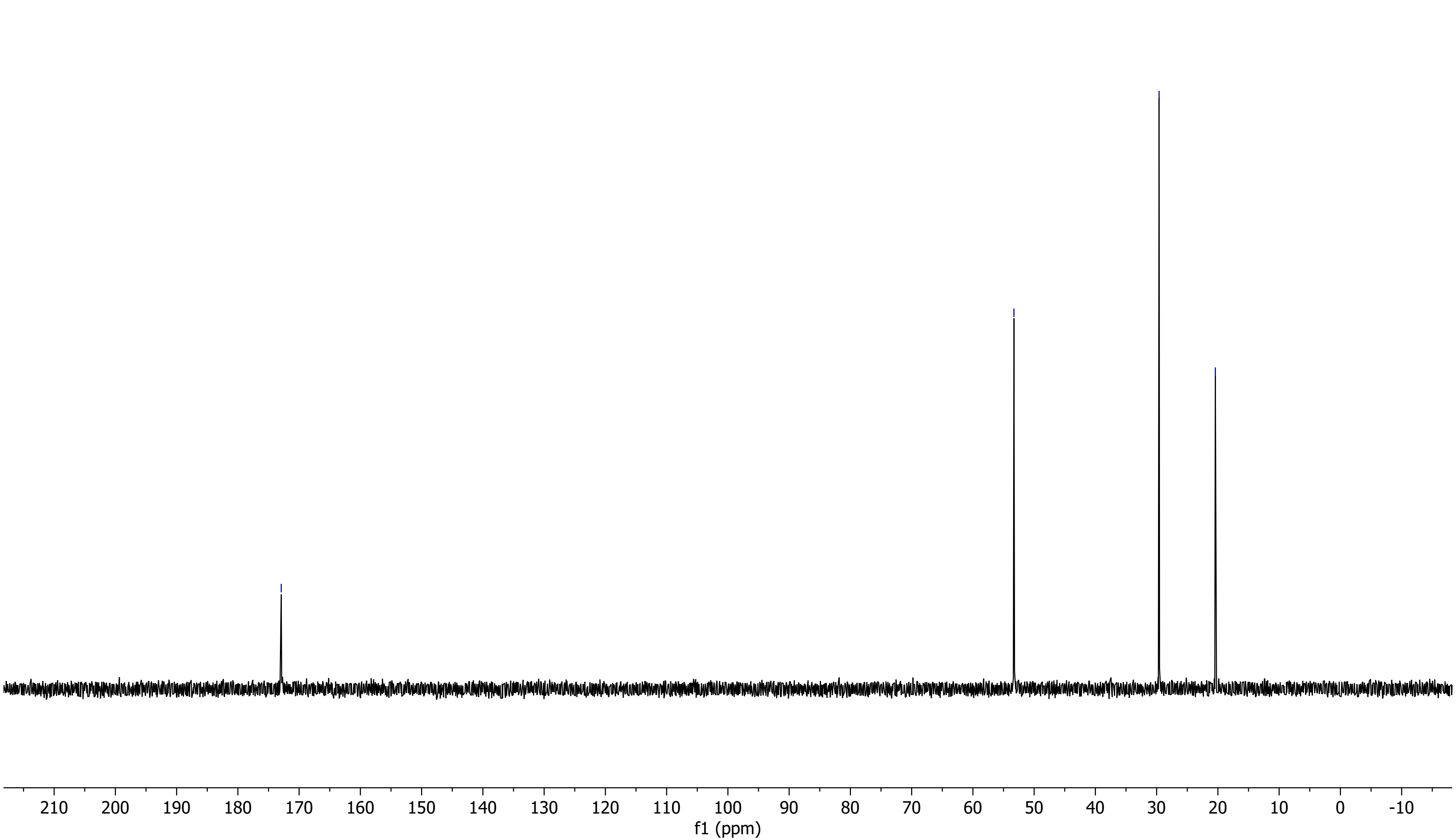

^{1}H NMR (400 MHz, $CDCl_3$)

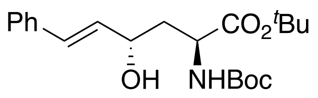

16

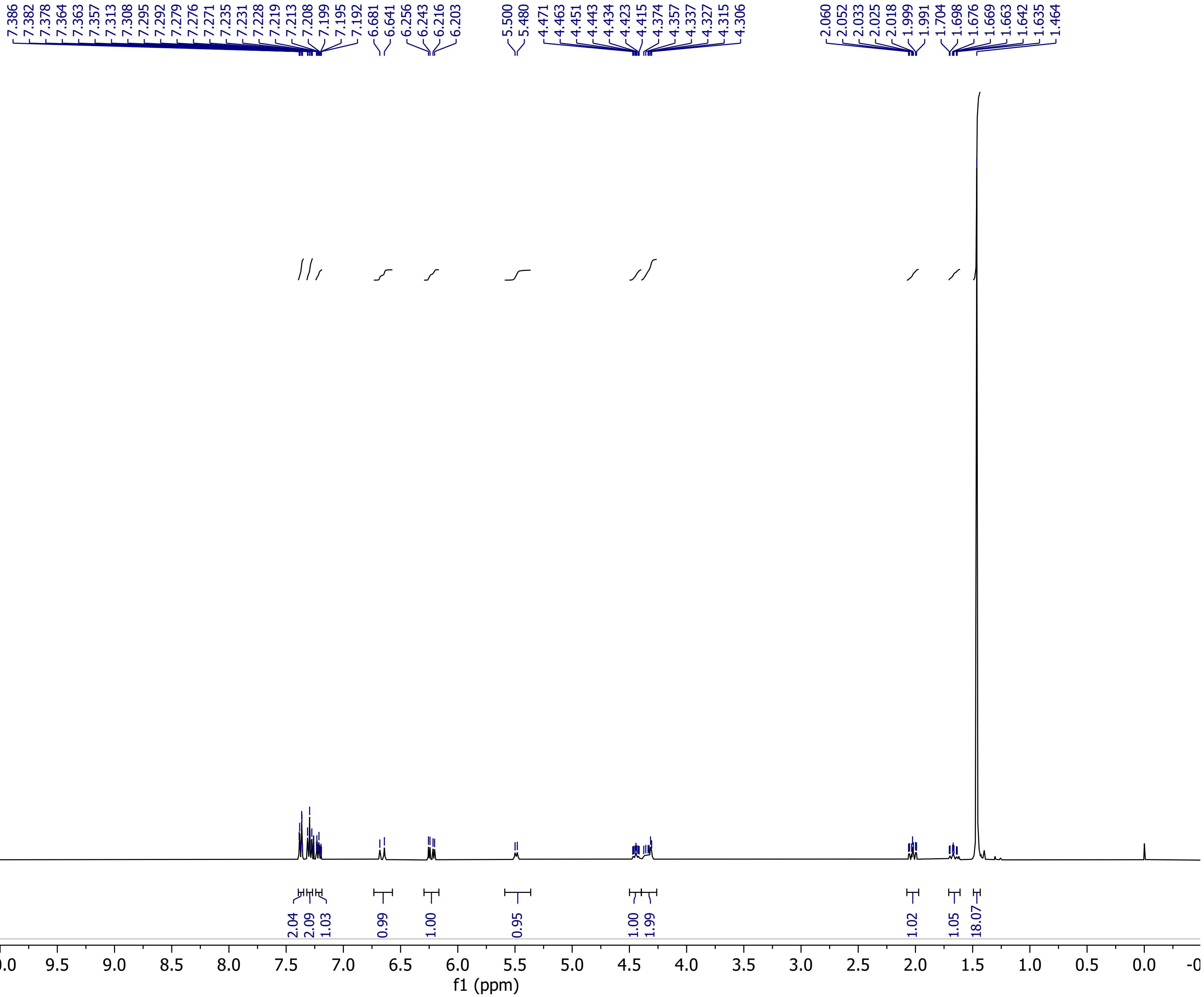

$^{13}C\{^{1}H\}$ NMR (101 MHz, $CDCl_3$)

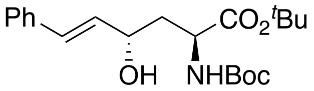

16

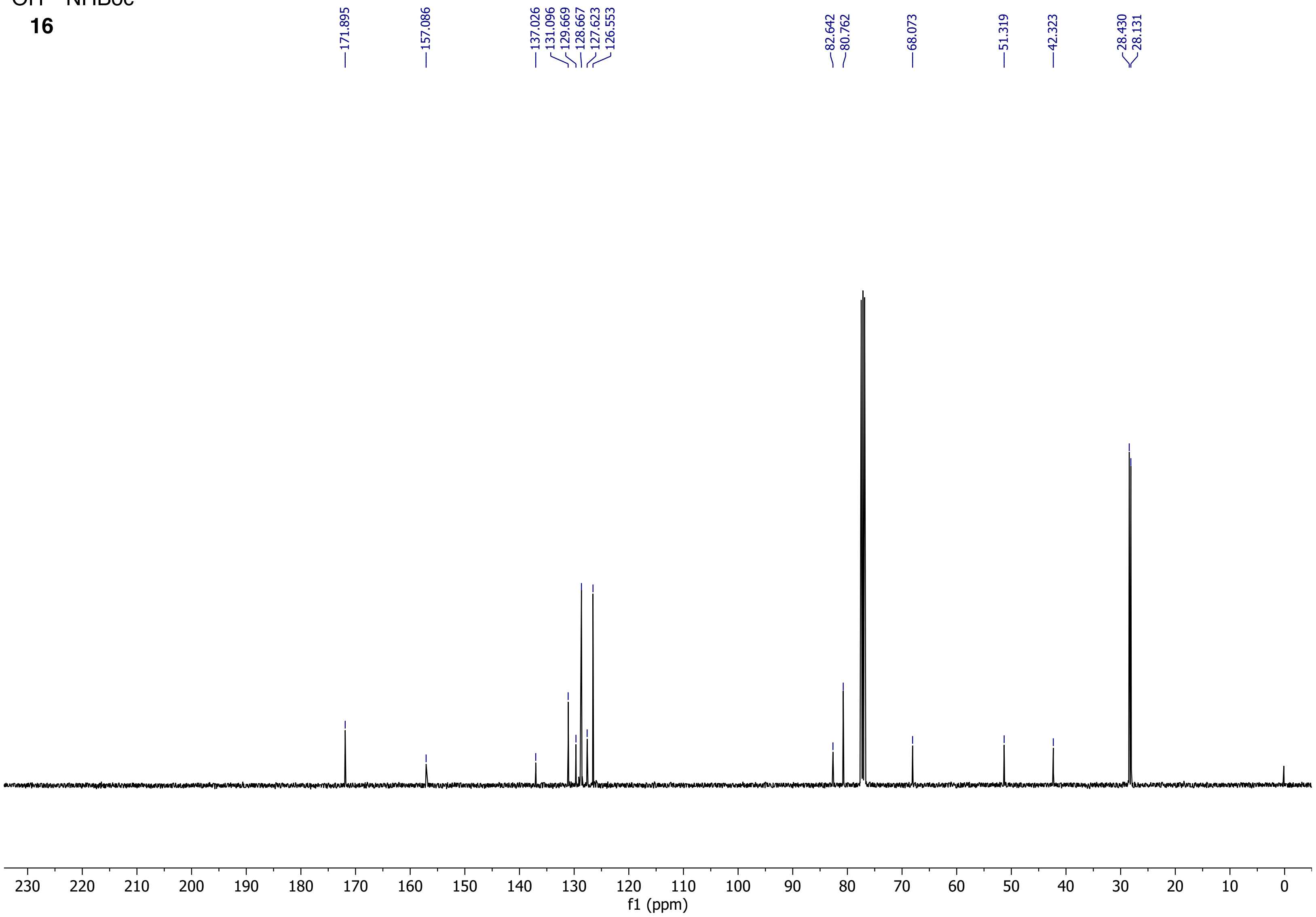

^{1}H NMR (400 MHz, $CDCl_3$)

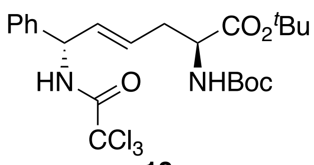

18

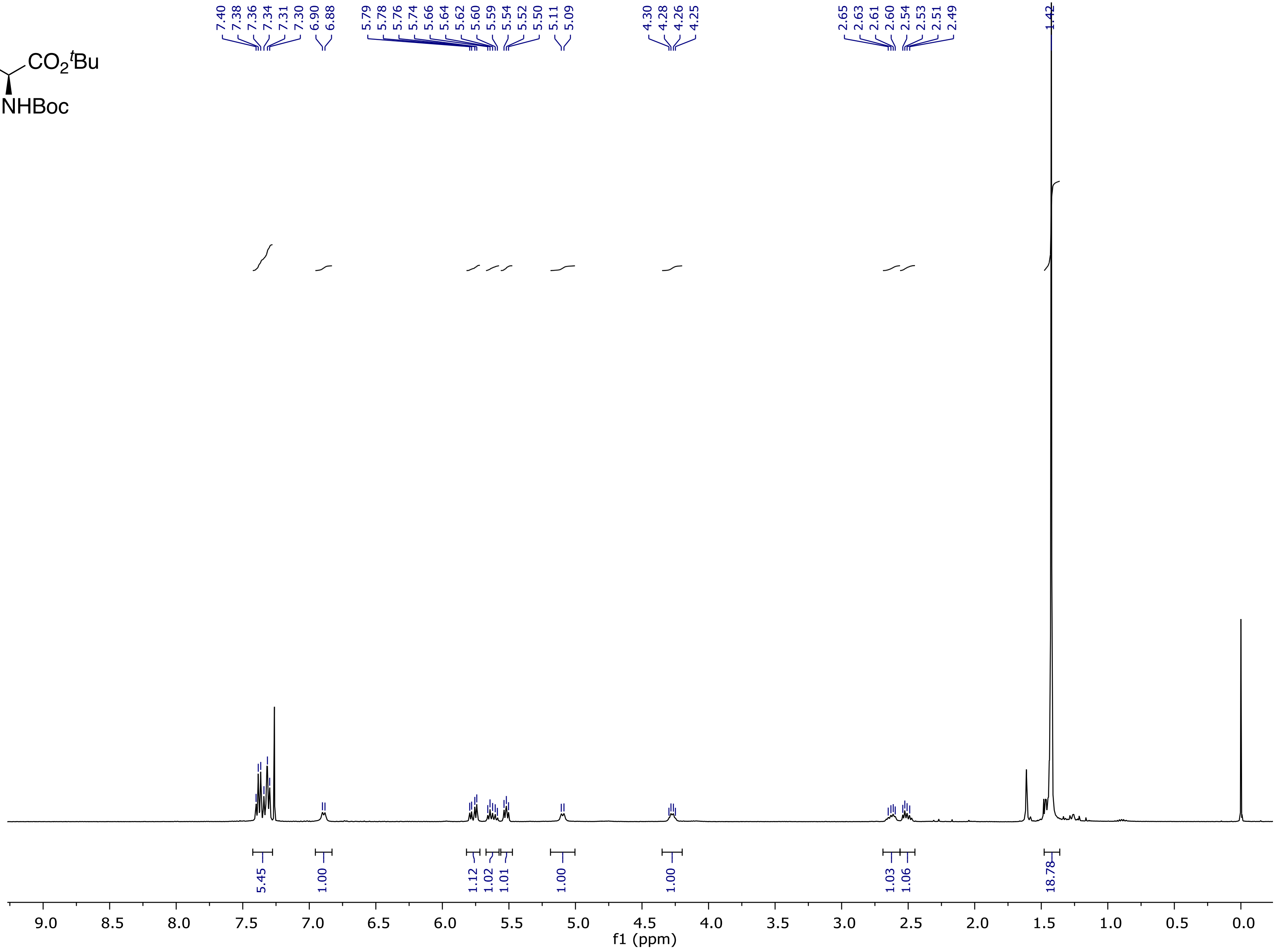

$^{13}C\{^{1}H\}$ NMR (101 MHz, $CDCl_3$)

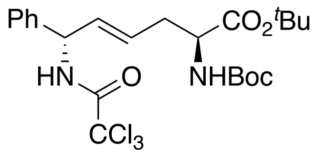

18

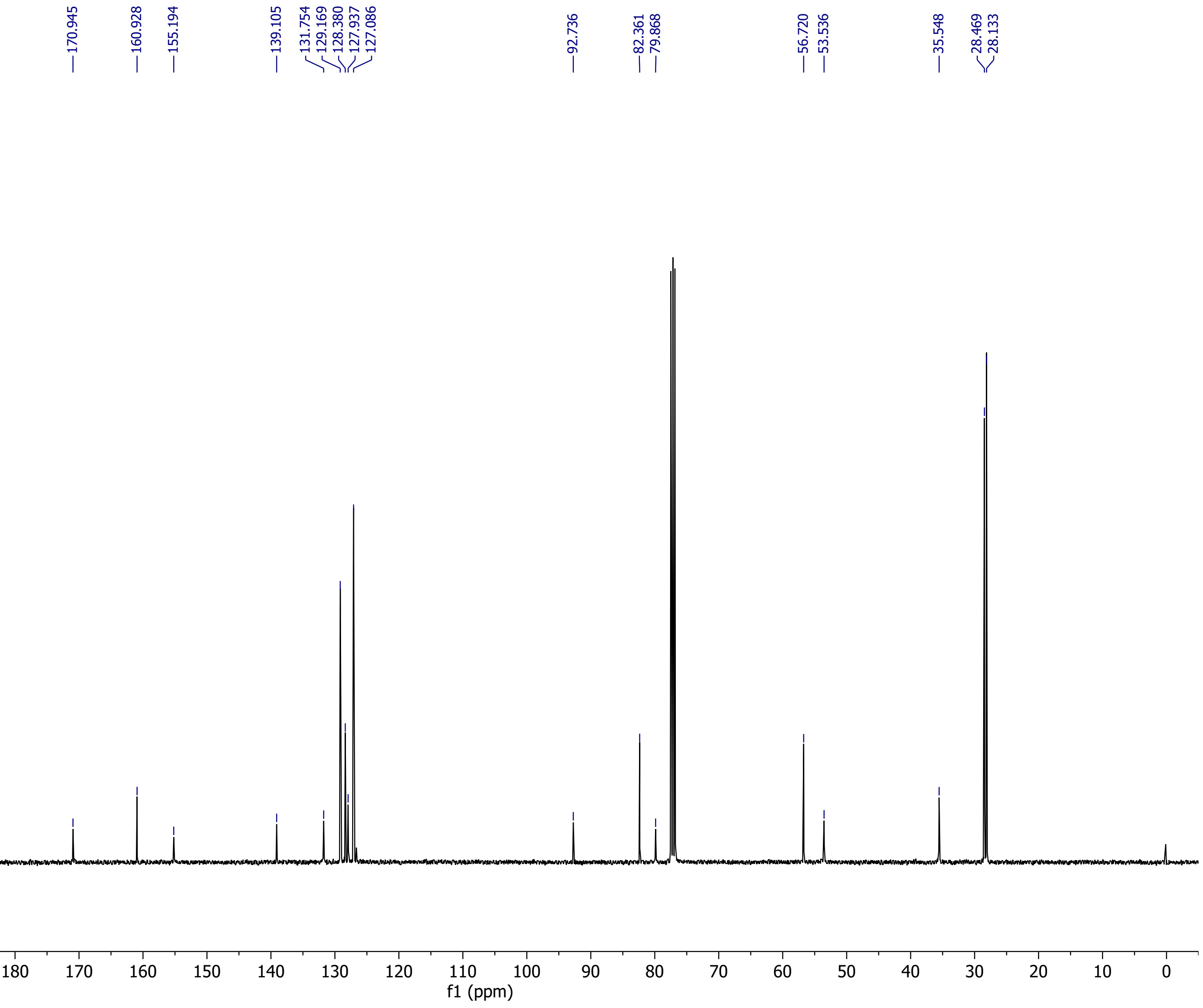

^{1}H NMR (400 MHz, $CDCl_3$)

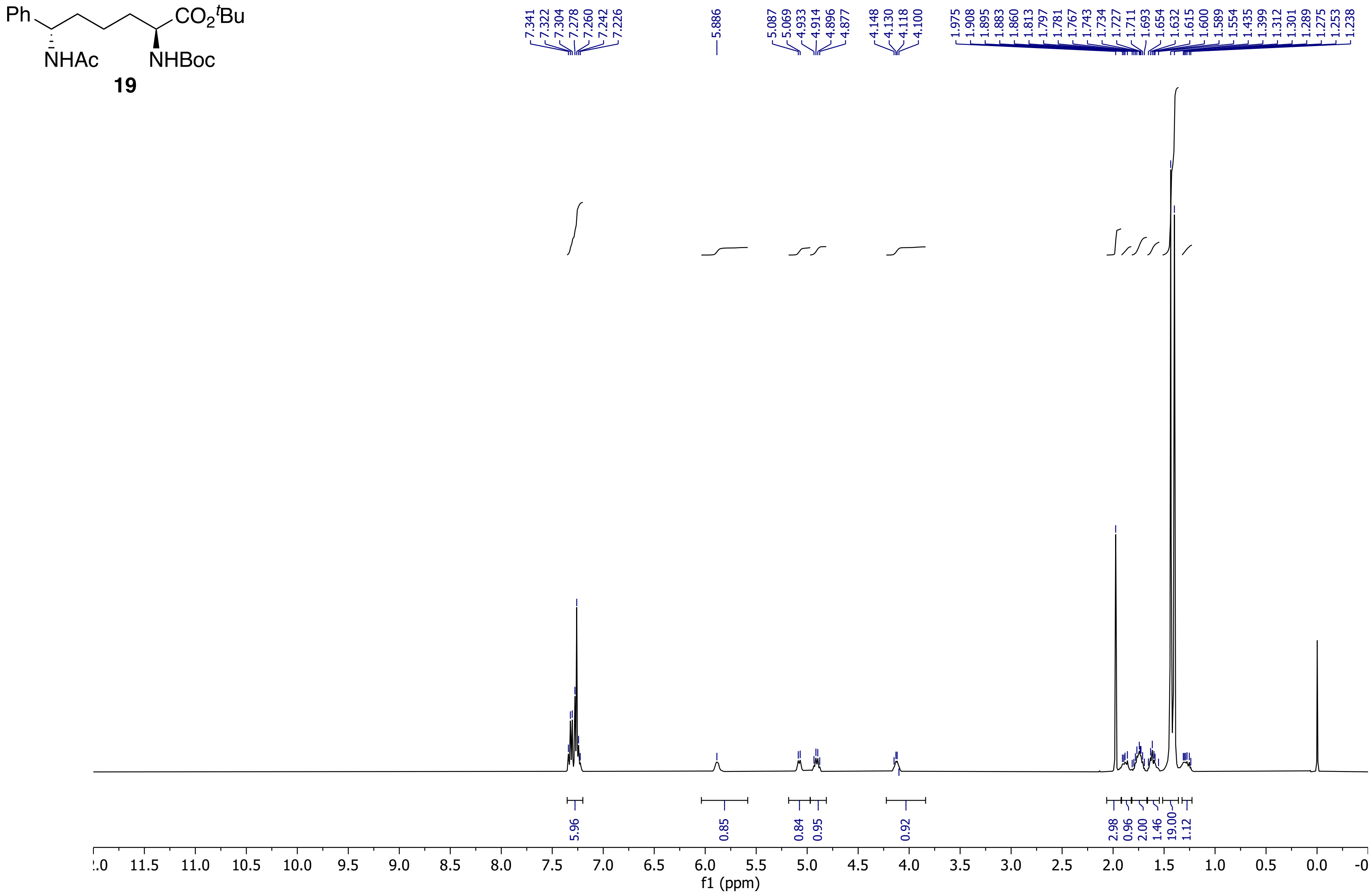

$^{13}C\{^1H\}$ NMR (101 MHz, $CDCl_3$)

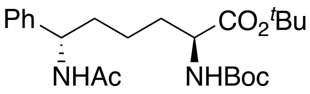
19

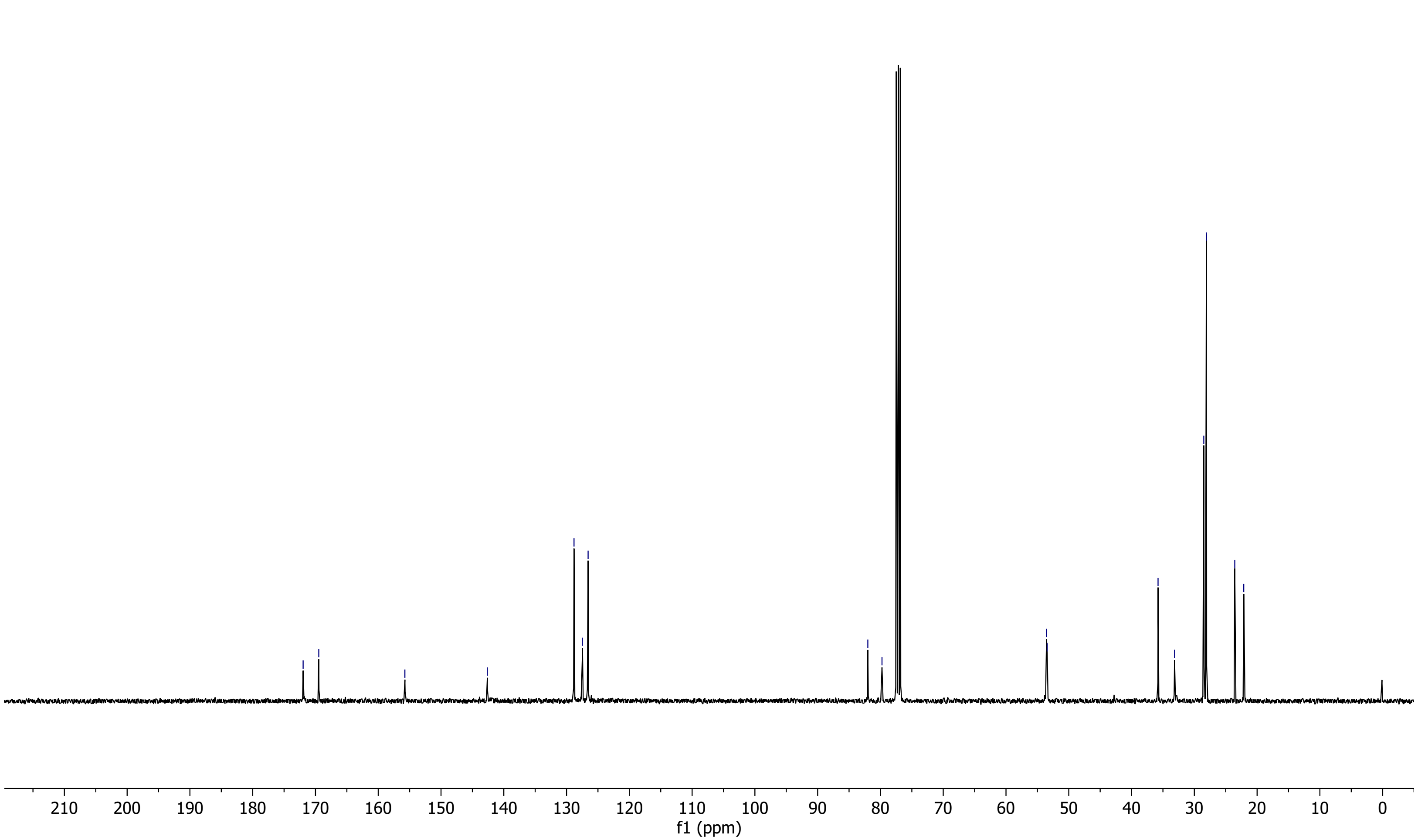

^{1}H NMR (400 MHz, $CDCl_3$)

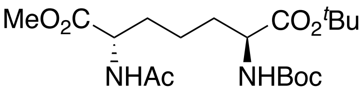

20

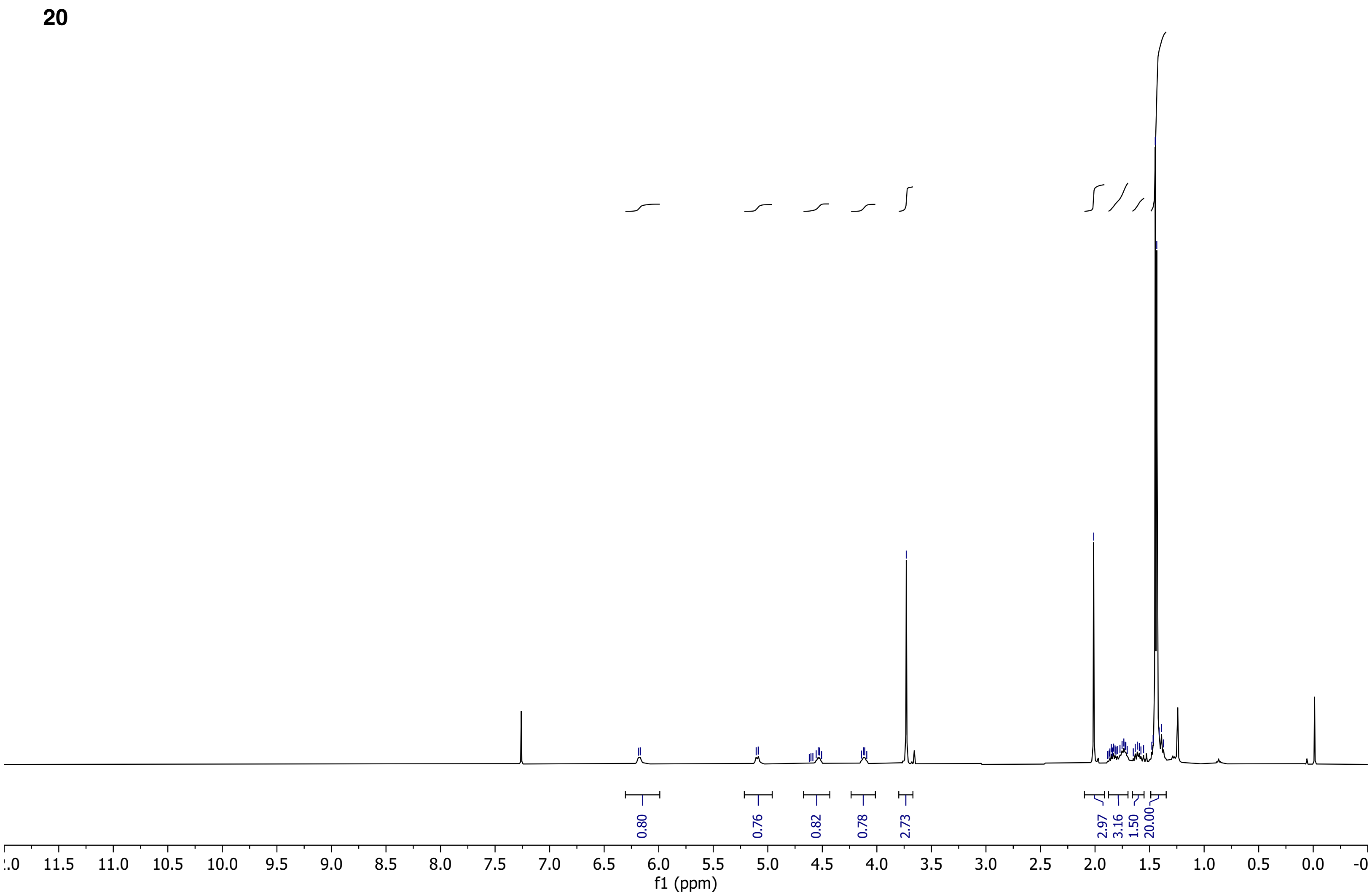

$^{13}C\{^{1}H\}$ NMR (101 MHz, $CDCl_3$)

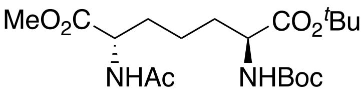

20

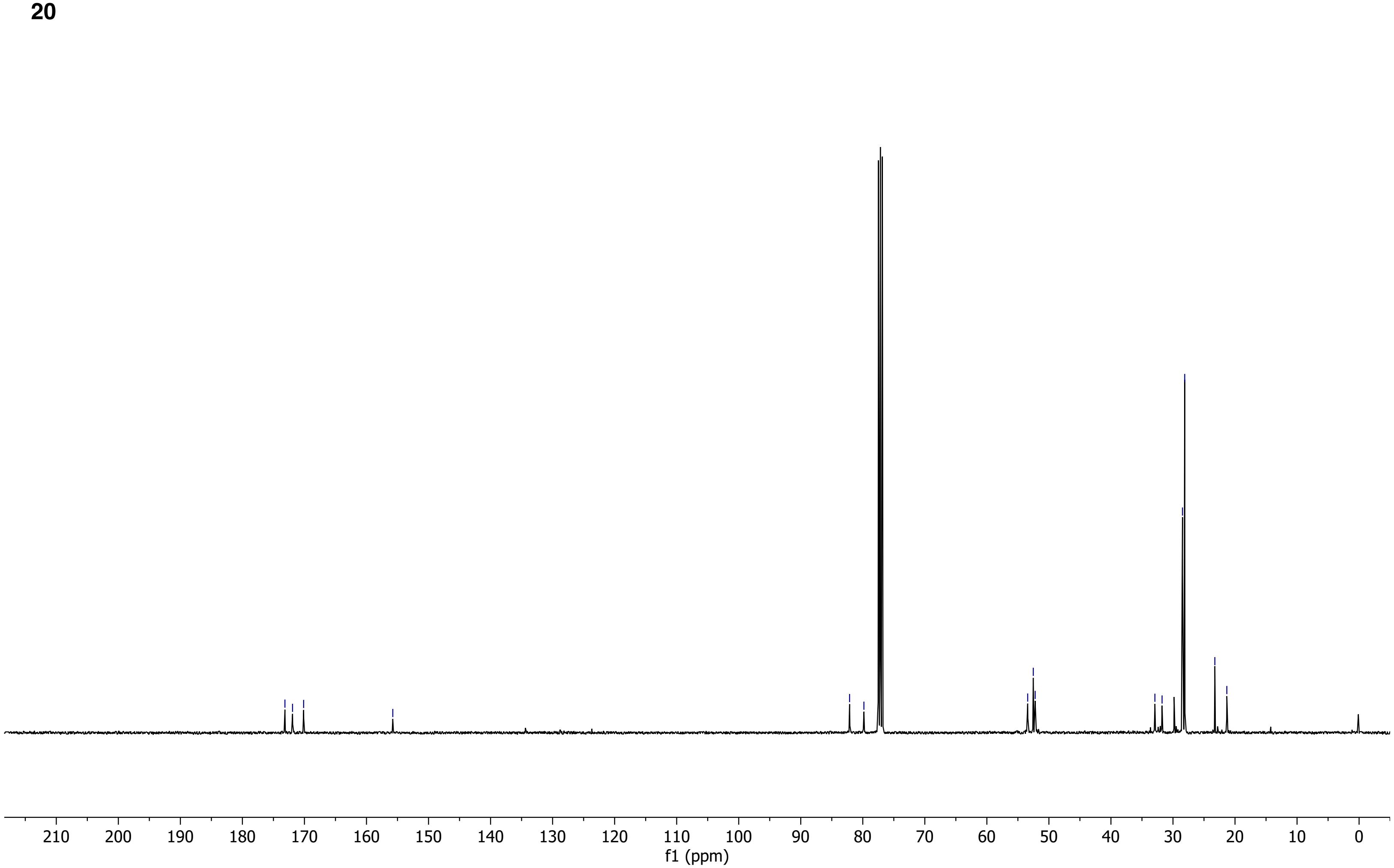

^{1}H NMR (400 MHz, CD_3OD)

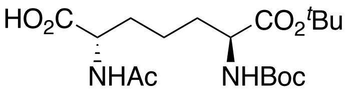

21

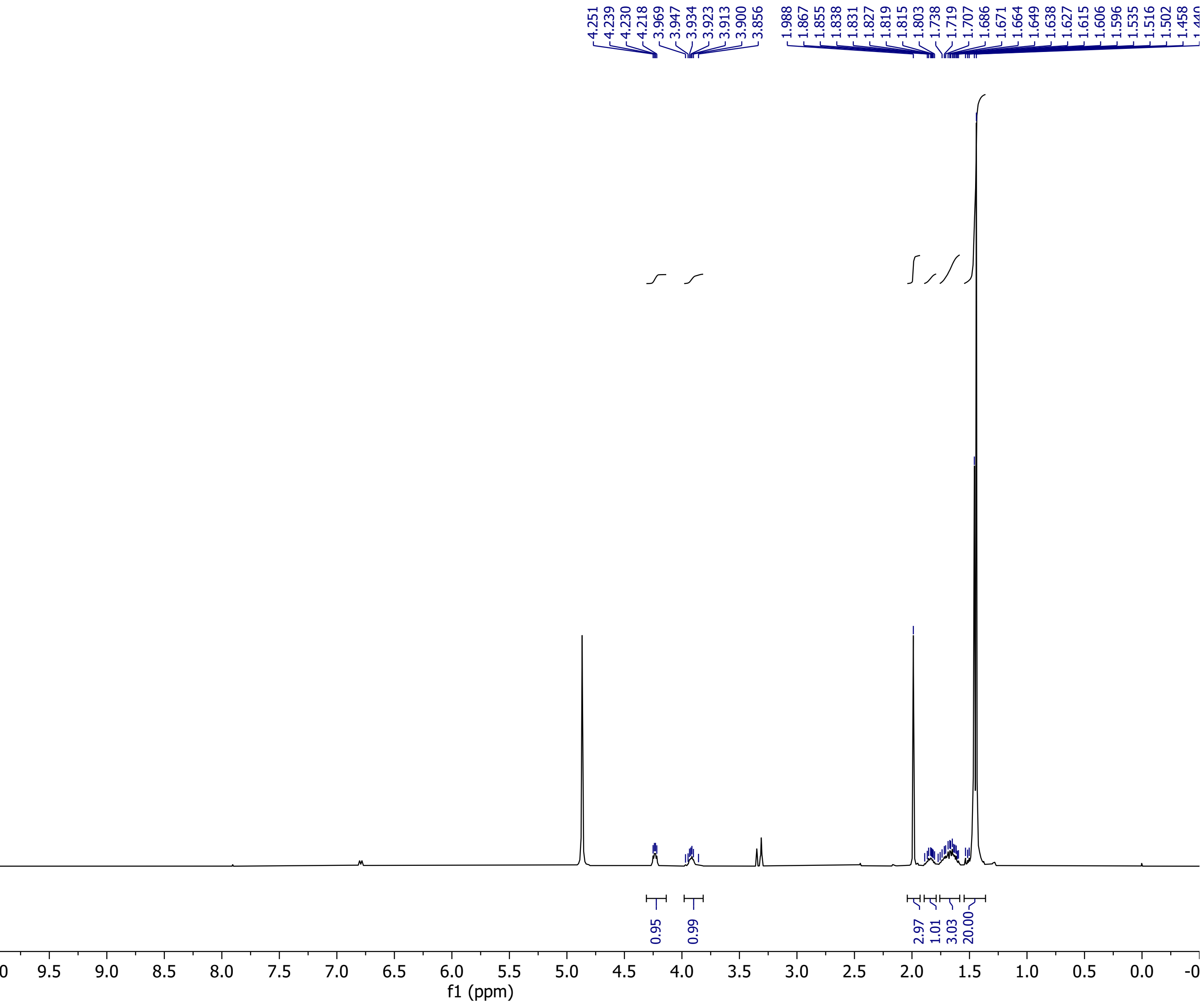

$^{13}C\{^{1}H\}$ NMR (101 MHz, CD_3OD)

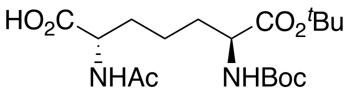

21

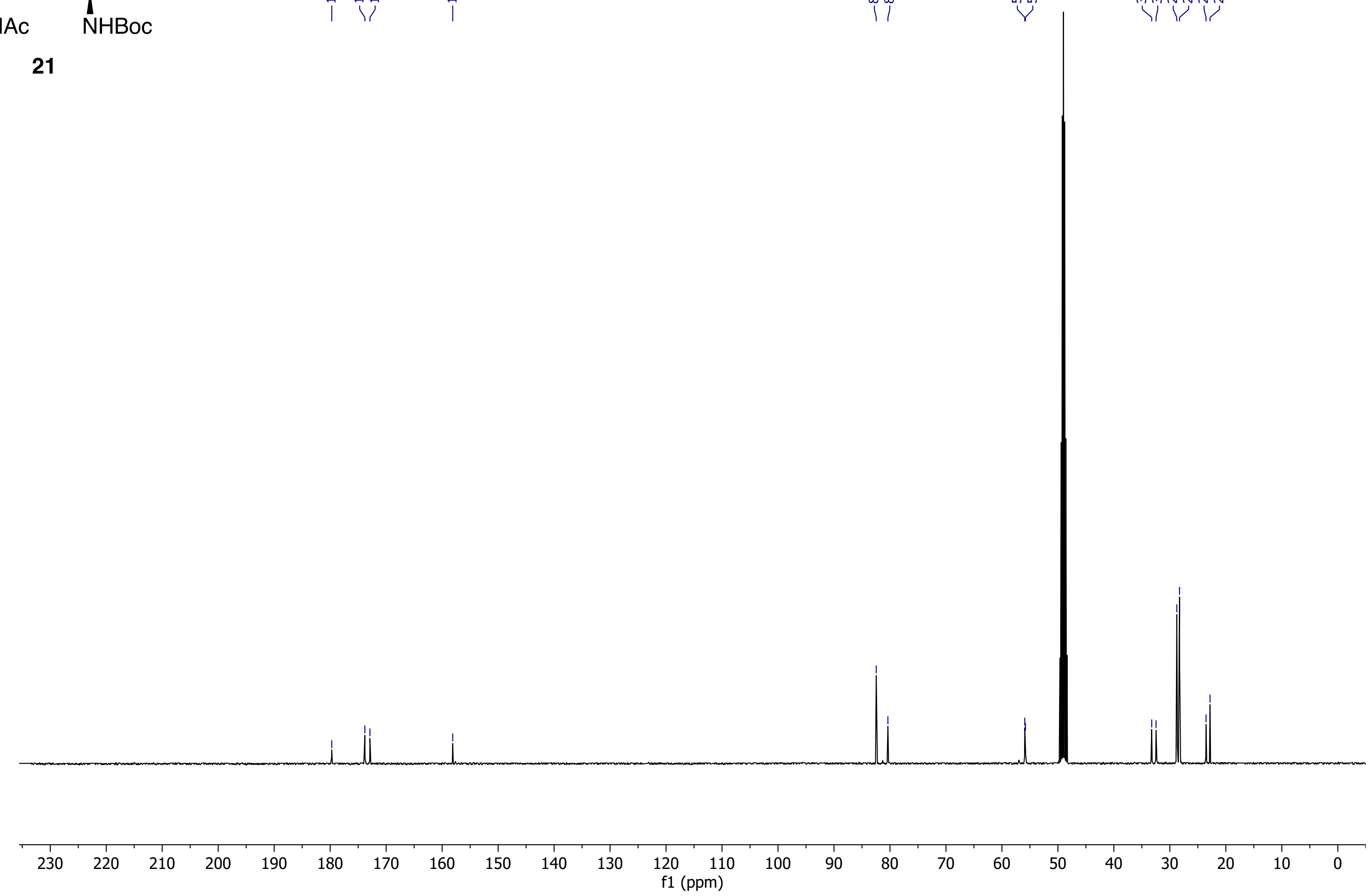

^{1}H NMR (400 MHz, D_2O)

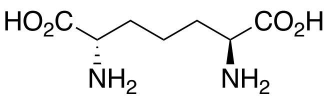

3

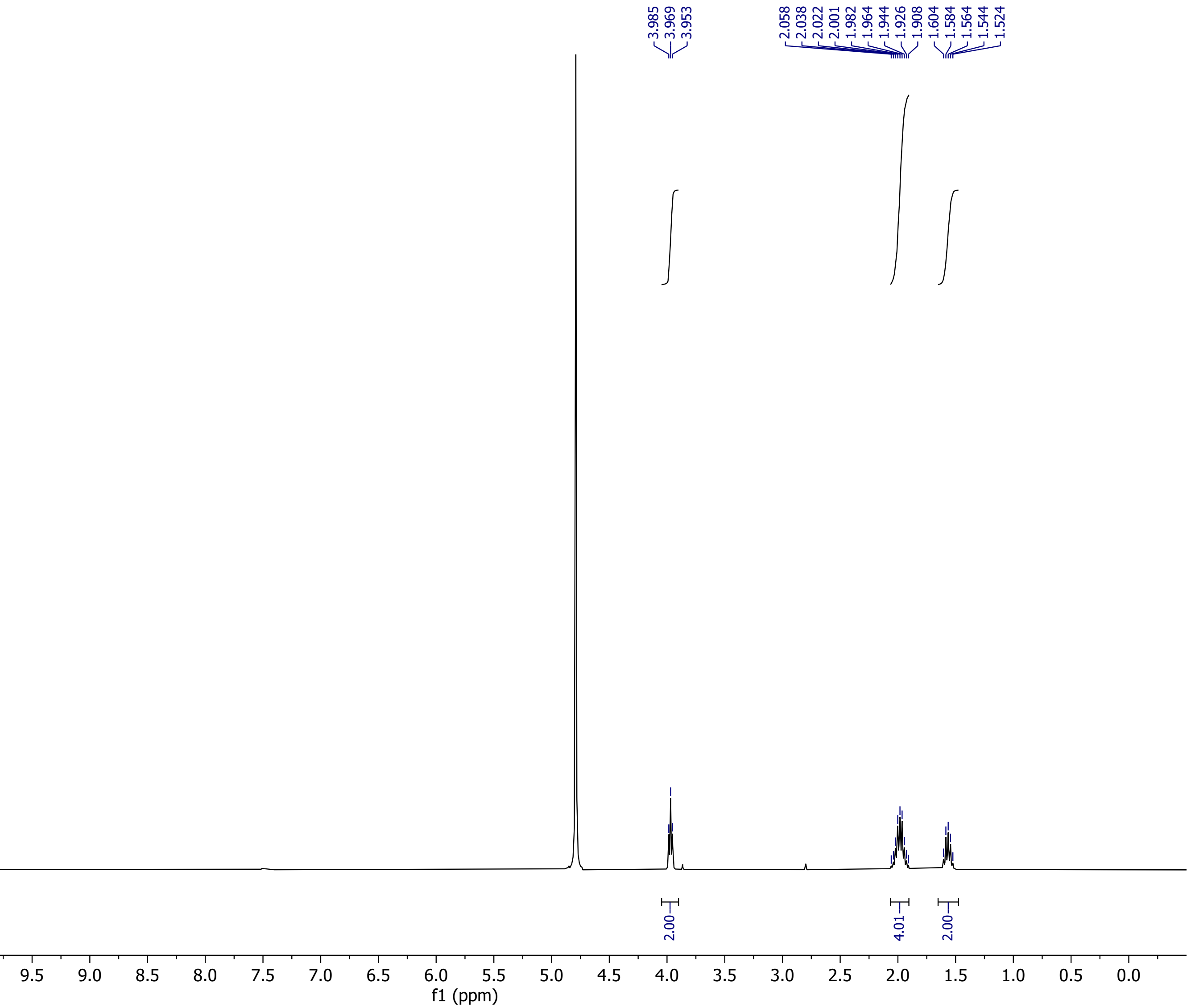

$^{13}C\{^1H\}$ NMR (101 MHz, D_2O)

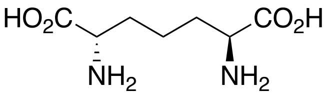

3

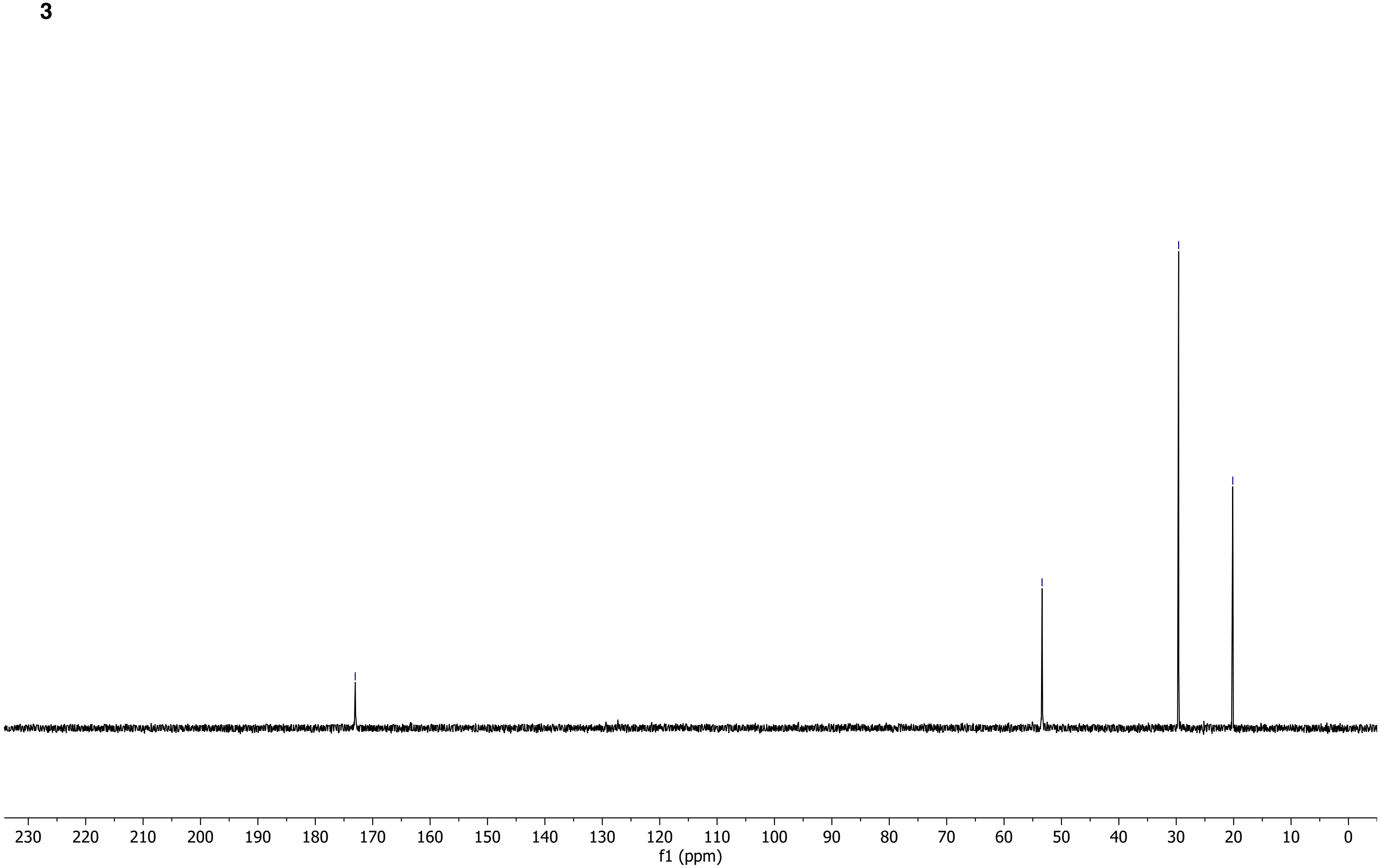

^{1}H NMR (400 MHz, $CDCl_3$)

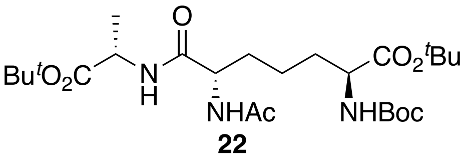

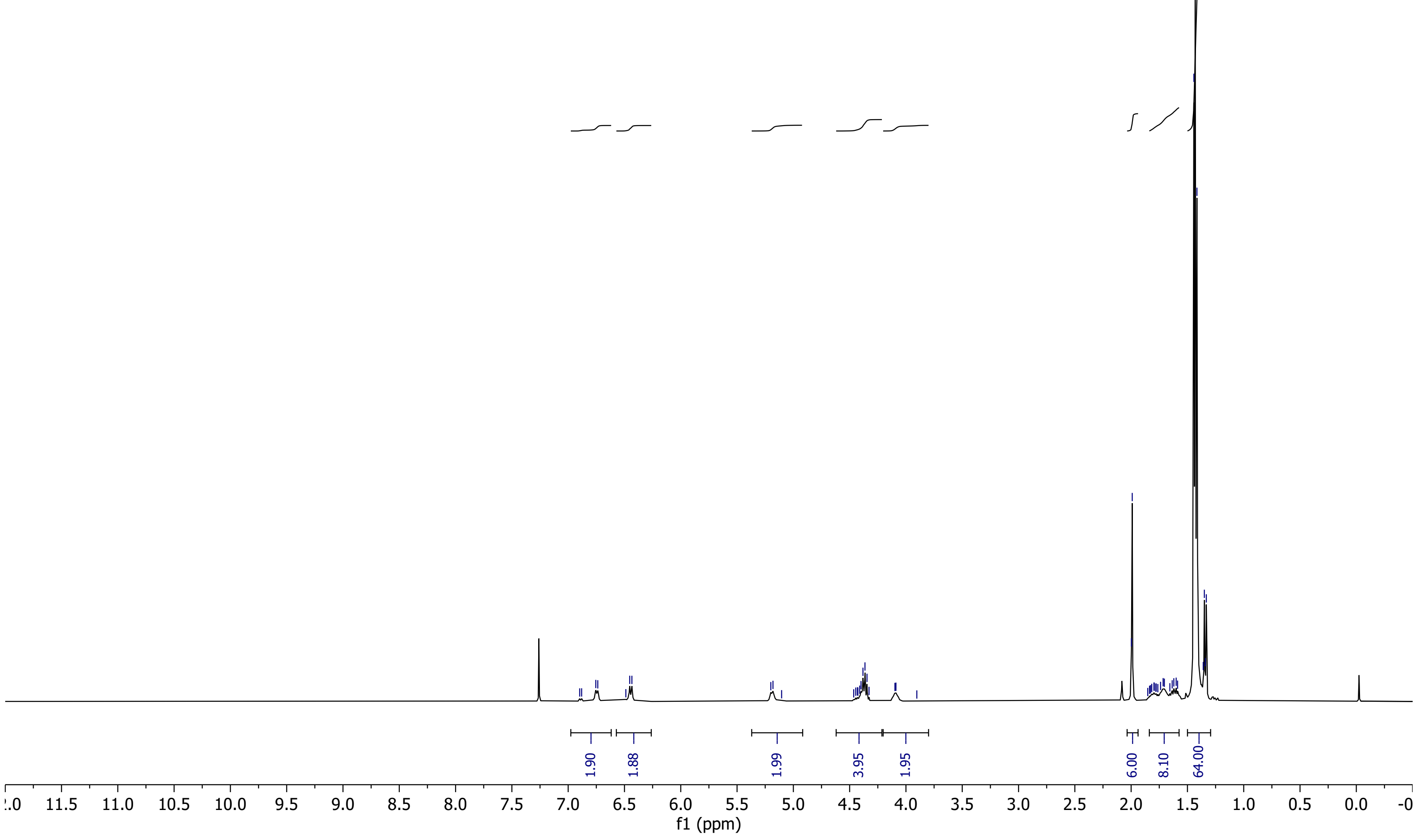

$^{13}C\{^{1}H\}$ NMR (101 MHz, $CDCl_3$)

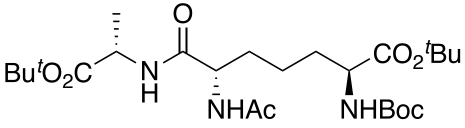

22

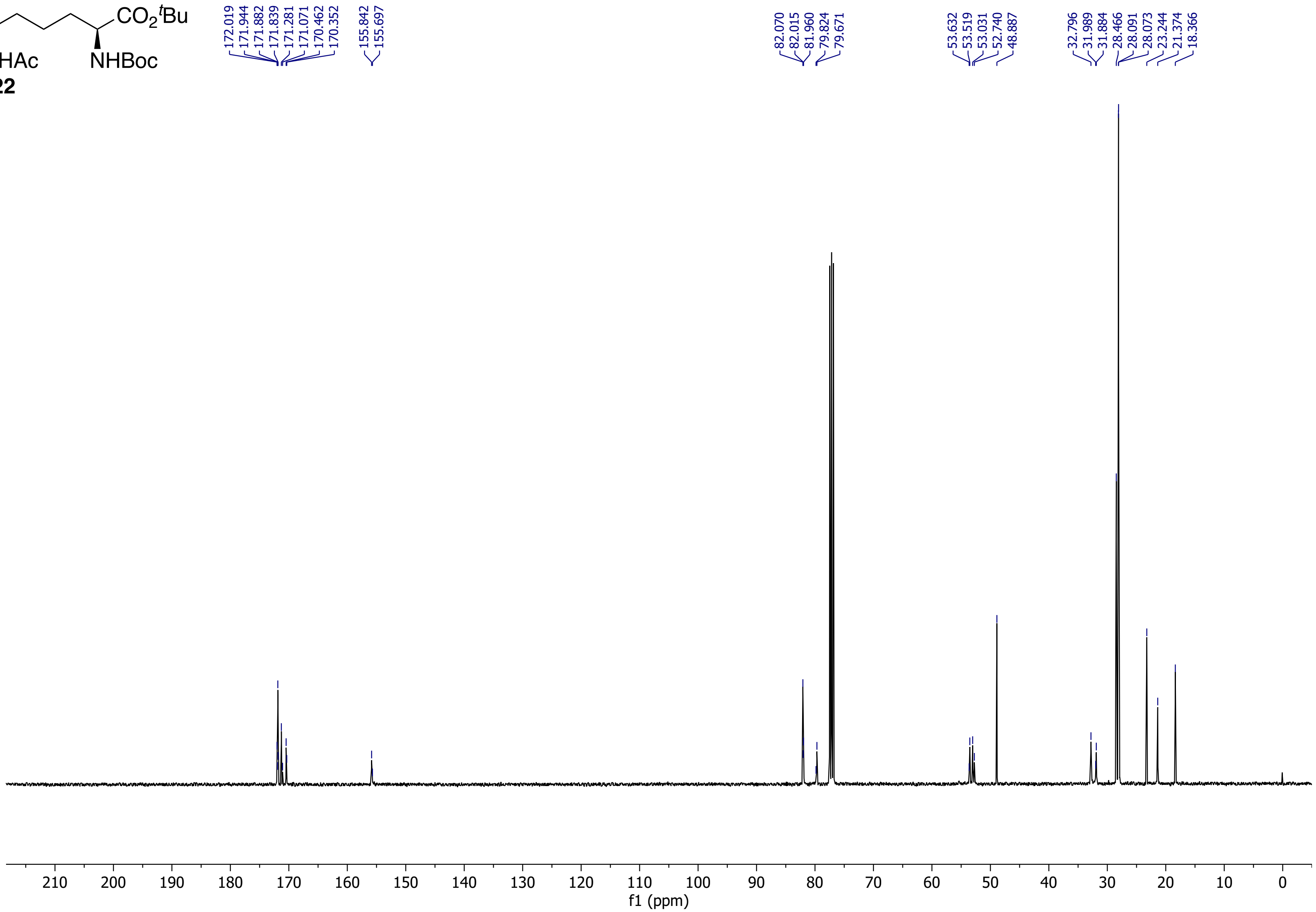

Supplement: Supplementary file 1 — jo4c00916_si_001.pdf [file jo4c00916_si_001.pdf]
